# Corporate Resonance of Food Safety Risk: A Space–Time Perspective

**DOI:** 10.3390/foods15162940

**Published:** 2026-08-21

**Authors:** Lei Wang, Tao Wang, Han Sun, Shuaibin Wang

**Affiliations:** 1 School of Economics and Management, Nanjing Tech University, Nanjing 211816, China; tao202561213277@njtech.edu.cn (T.W.);; 2 School of Economics and Finance, South China University of Technology, Guangzhou 510006, China

**Keywords:** corporate resonance, food safety risk, space–time perspective, cellular automata, epidemic model

## Abstract

Employing a space–time perspective, this study develops a CA-SHIRS model of corporate resonance diffusion of food safety risk, drawing on complex network theory and cellular automata theory. The study then examines the mechanisms and spatial–temporal evolution characteristics of this diffusion, considering the interplay among food firm heterogeneity, media communication strategy, and government regulatory strategy. The study reaches the following conclusions: (1) Higher probabilities of infection, conversion, and immune failure speed up risk transmission within the spatial–temporal association network of food firms. By contrast, raising the immune probability and direct immune probability helps contain the scale of risk spread. (2) The intensity of corporate resonance diffusion is positively correlated with corporate influence and media influence, and negatively correlated with corporate social responsibility, media information disclosure intensity, government penalty intensity, and government regulatory information transparency. It exhibits an inverted U-shaped relationship with corporate risk preference and a positive U-shaped relationship with media reporting preference. (3) Both corporate influence and media influence reinforce corporate resonance diffusion, while government regulation effectively mitigates it. Firms with moderate risk preference are most significantly affected by extreme media coverage; corporate social responsibility and media information disclosure intensity can jointly suppress diffusion at these nodes. Government regulation exerts a stronger inhibitory effect on corporate resonance diffusion than the amplifying effect exerted by media communication.

## 1. Introduction

Food safety risk is broadly defined as the probability of health damage from foodborne hazards, but it also encompasses systemic failures in production, logistics, and information transmission that can trigger adverse behavioral responses among food firms through supply chain linkages and market interdependencies [1,2,3]. Its importance lies not only in public health outcomes, but also in its potential to destabilize food markets, disrupt supply chain operations, erode consumer confidence, and induce collective risk resonance among firms. Given its global significance, food safety risk has attracted considerable worldwide attention, and a fairly comprehensive body of laws has been established to address it. In international law, the Codex Alimentarius framework (FAO/WHO) provides internationally recognized reference standards for food safety. At the national level, the Food Safety Law of the People’s Rep. of China establishes the domestic regulatory framework. At the regional level, the E.U. General Food Law (Regulation (EC) No. 178/2002) sets out binding principles of risk analysis, traceability, transparency, and operator accountability. Corporate resonance, in the context of food safety risk, refers to the synchronized behavioral responses and risk perceptions among food firms triggered by a food safety incident, propagating through supply chain linkages, capital ties, regional market competition and cooperation, and information exchange networks [4,5,6]. This concept is distinct from risk contagion, spillover effects, and crisis diffusion. Risk contagion is the stepwise transmission of risk through direct or indirect channels of interconnection. Spillover effects are externalities on third parties. Crisis diffusion emphasizes the spread of crisis consequences through interdependent networks across geographical or sectoral dimensions. Corporate resonance instead captures the bidirectional, multi-layered, and self-reinforcing amplification of risk among firms. The food industry’s high supply chain integration, information asymmetry, and consumer sensitivity make this perspective particularly relevant for capturing emergent interactive dynamics beyond linear frameworks.

Over the past several years, several grave food safety incidents have taken place. One such case is the 2025 caesium-137 contamination of Indonesian prawns destined for the US market, which posed a serious threat to consumer health and safety while quickly spreading across time and space. This has triggered a systemic crisis of confidence in food firms and caused turmoil in the food market [7]. The diversification of information transmission channels is accelerating, especially driven by the rapid growth of social networks and media platforms. As a result, the spatial–temporal evolution of food safety risk has grown increasingly intricate. This pattern is observable in the stepwise diffusion of food safety risk, starting from individual food firms, then extending to the whole industry, and eventually reaching a global scale. Moreover, as food safety risk incidents diffuse, the behavioral responses of food firms display a resonance effect that extends beyond individual firms, time, and geographical boundaries. The transmission of risk between food firms often far exceeds the actual impact caused by a single incident. In addition to individual factors within food firms, the corporate resonance of food safety risk is also affected by external forces, including supply chain networks, media communication, and government regulation [8,9,10]. Consequently, studying the mechanisms of corporate resonance in the diffusion of food safety risk and their spatial–temporal evolution from a space–time perspective is important. It holds significant practical implications for maintaining production order within the food industry and ensuring the stability of food markets.

A large body of literature now exists on food safety risk. Researchers have investigated multiple dimensions, including risk perception of food firms [11], media coverage [12,13], and government regulation [14,15]. Prior studies suggest that the food industry now exhibits new features, including decentralized operations, large-scale distribution across multiple formats and channels [16]. Consequently, as food safety risk diffuses spatially, this resulting corporate resonance will intensify both the severity and the reach of such incidents [17,18]. Adopting a space–time perspective, the corporate resonance of food safety risk manifests not solely in the risk cognition and reactive decisions of single food firms, but likewise in the multitiered and evolving interactions among clusters of such firms as these dynamics unfold across both temporal and spatial scales [19]. In particular, as information communication speeds up and inter-firm networks expand, this resonance surpasses temporal and spatial limits. Consequently, it inflicts serious adverse effects on both the supply chain for food and the wider food market [12,20]. Prior research, however, has largely restricted its focus to either a purely temporal or a purely spatial perspective, typically examining isolated factors such as media communication or government regulation. In doing so, they have neglected the behavioral characteristics of food firms as the principal actors in risk assumption and propagation. Moreover, they have neglected the impact of the interactions among food firms, the media, and the government on resonance diffusion. Consequently, this study adopts a space–time perspective and comprehensively considers interactions among food firm heterogeneity, media communication strategy, and government regulatory strategy. The aim is to explore in depth the mechanisms of this resonance diffusion and their spatial–temporal evolution characteristics.

Complex network-based epidemic models have found application across a wide range of areas, including online public sentiment [21,22], computer viruses [23,24], risk contagion in supply chains [25,26], and food safety risk [13,27]. Taking the study of Trazias et al. [27] as an example, this team employed epidemic models to investigate the effects of physiological behaviors of humans and dairy cattle on the transport of environmental pollutants. Their simulations show that salmonellosis transmission depends on both contaminated dairy products and environmental factors, revealing distinct features and regularities. However, current applications of epidemic models to the propagation of food safety risk have primarily focused on basic models such as SI, SIS and SIRS, with insufficient exploration of the complex dynamic transitions in the infection status of food firms during risk propagation, as well as the intricate interactions between firms across various states. The corporate resonance diffusion of food safety risk displays distinct characteristics in its spatial–temporal evolution [13]. Cellular automata (CA), in addition, are grid-based dynamical models which perform discretization of time, space, and state. They display significant spatial–temporal evolutionary characteristics and can reproduce the dynamic behaviors of complex systems by applying specific transition rules [28,29]. Hence, the integration of cellular automata and epidemic models provides an effective means to explore the spatial–temporal evolution of corporate resonance of food safety risk. Building on the SHIRS epidemic model, this study introduces cellular automata theory to optimize the rules governing the corporate resonance diffusion of food safety risk. Such an integration both refines traditional infectious disease transmission models and addresses the interactions and feedback loops across multiple levels among food firms across spatial–temporal dimensions.

In summary, this paper takes a space–time perspective. It incorporates the interactive effects of food firm heterogeneity, media communication strategy, and government regulatory strategy. It then builds a CA-SHIRS model of corporate resonance diffusion of food safety risk. This model is developed using cellular automata and the SHIRS epidemic model. Subsequent numerical simulations are conducted to examine how corporate resonance diffusion of food safety risk evolves across space and time. Specifically, this study addresses three research questions: (1) What are the dynamic evolutionary patterns of corporate resonance diffusion within the spatial–temporal association network of food firms? (2) How do food firm heterogeneity, media communication strategy, and government regulatory strategy individually influence this diffusion? (3) How do their interactions shape the spatial–temporal evolution of this diffusion? To answer these questions, this study aims to construct a CA-SHIRS model, examine the individual effects of each factor, and reveal the interactive mechanisms among the three dimensions. We highlight three main contributions of this paper in what follows. (1) A perspective, either purely temporal or purely spatial, has been typically adopted by existing studies. They usually focus solely on isolated factors such as media communication or government regulation. By contrast, this paper takes a space–time perspective to examine the spatial–temporal evolution characteristics of corporate resonance diffusion of food safety risk, specifically under the interaction of food firm heterogeneity, government regulatory strategy, and media communication strategy. (2) The present study differs from conventional basic infectious disease dynamical models by integrating cellular automata theory into the epidemic model. Such integration achieves two objectives. First, it refines the diffusion rules for the corporate resonance of food safety risk. Second, the CA-SHIRS model developed here enables a more faithful depiction of how food firms locally interact and are spatially dispersed. (3) The present study has produced a number of conclusions that are both novel and practically applicable. The interplay between corporate influence and media influence produces a dual-amplifying effect on the intensity of corporate resonance diffusion of food safety risk. Meanwhile, government regulatory strategy effectively mitigates this effect. Firms with moderate risk preference are most significantly affected by extreme media coverage. Moreover, government regulation exerts a stronger risk-suppressing influence than the risk-amplifying influence of media communication.

## 2. Theoretical Background

**Figure 1:**
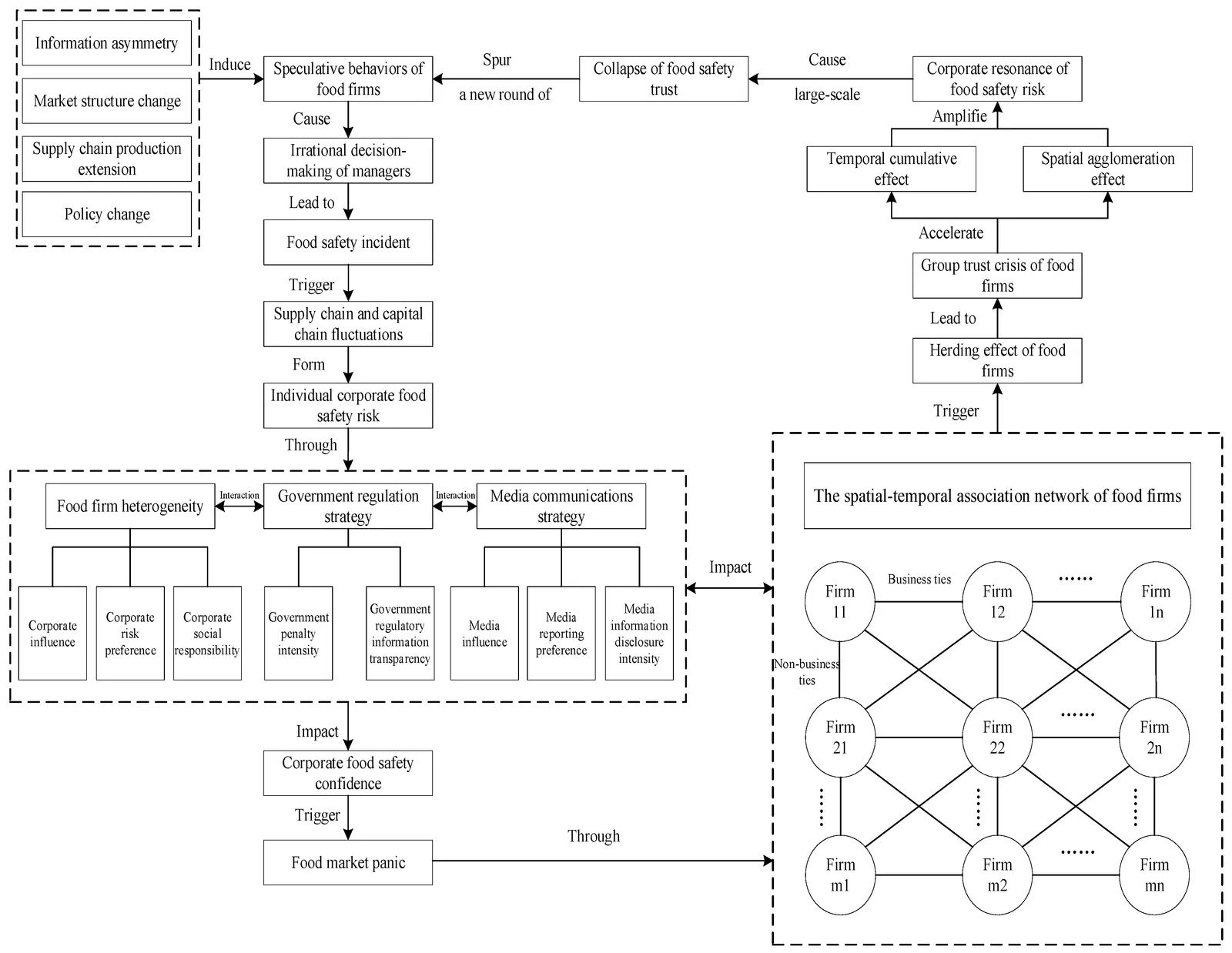
Mechanism of corporate resonance diffusion of food safety risk under the interaction of food firm heterogeneity, government regulatory strategy and media communication strategy.

Corporate resonance in food safety risk, viewed from a space–time perspective, refers to a phenomenon in which food firms, subsequent to a food safety incident, construct a network of spatial–temporal connections within a particular temporal and spatial setting. This network is built through business and non-business linkages, supply chain cooperation, regional market competition and cooperation, and information exchange. This network facilitates a synchronous linkage between risk perception and behavioral responses, triggering a collective risk oscillation across the food sector [12]. At the core of corporate resonance within food safety risk is that individual food firms cannot always absorb food safety risk internally. Instead, these risks evolve into collective behavioral resonance across firms, time, and geographical regions. This occurs through their spatial–temporal association network, under multiple external triggers such as information asymmetry, changes in market structure, the extension of supply chain production, and policy shifts [30,31]. The interaction of food firm heterogeneity, media communication strategy, and government regulatory strategy influences this resonance process. Over time, it displays cumulative effects, as well as dynamic features of spatial clustering and diffusion. Figure 1 presents the mechanism of corporate resonance diffusion of food safety risk under the interaction of food firm heterogeneity, government regulatory strategy, and media communication strategy.

Speculative behavior among food firms, induced by market information asymmetry and the extension of supply chains, gives rise to the corporate resonance of food safety risk [14]. Some food firm managers make irrational decisions driven by short-term gains, leading to the occurrence of food safety incidents. Once an incident erupts, food safety risk signals are transmitted along two pathways. First, through media communication strategy, such as emotional reporting by certain media outlets and synchronized cross-platform communication, the signals are rapidly amplified. This damages the market reputation of food firms and erodes consumer confidence. Second, through government regulatory strategy, such as adjustments to the severity of penalties and the disclosure of regulatory information, firms’ compliance expectations and risk perceptions are influenced. At this stage, the heterogeneity of food firms begins to play a role. Food firms with significant influence see their food safety risk signals radiate widely. Those with moderate risk preference are more likely to follow the trend and become part of the mainstream. Meanwhile, those with a strong sense of social responsibility may proactively suppress the spillover of risks.

Rather than proceeding along a linear path, the diffusion of food safety risk emerges as a networked event through the spatial–temporal association network of food firms. These linkages encompass supply chain partnerships, regional market competition and cooperation, financial connections, and information exchange [32,33]. Once a food firm at a particular node within the network, particularly a highly connected core firm, is exposed to risk or experiences a loss of trust, this will affect its associated food firms through channels such as commercial transactions, public opinion dynamics, and capital withdrawal. Under the influence of the herd effect, associated food firms may adopt imitative retrenchment, suspend cooperation, or overreact in an attempt to avoid losses or pursue short-term safety, thereby triggering a crisis of trust within the food firm community [34]. At this stage, the risk is no longer confined to a single incident but evolves into systemic panic within the food industry. This process exhibits distinct spatial–temporal evolutionary characteristics. In the temporal dimension, food safety risk signals accumulate over time, with the collective risk memory and vigilance of food firms continuously increasing, leading to an amplification of the impact of subsequent similar incidents. In the spatial dimension, food safety risk forms a spatial clustering effect along the spatial–temporal association network of food firms, erupting first in core segments of the industrial chain, regional industrial clusters, or capital-intensive sectors, and gradually spreading to peripheral segments and outlying regions. Ultimately, a large-scale collapse of trust in food safety will in turn spur a new round of speculative behavior among food firms. Some food firms may adopt more short-sighted strategies out of desperation, thereby creating a negative cycle of food safety risk regeneration that continuously intensifies the strength and scope of corporate resonance regarding food safety risk.

## 3. Materials and Methods

### 3.1. The CA-SHIRS Model of Corporate Resonance Diffusion of Food Safety Risk

#### 3.1.1. Definition of Cellular States

A cellular automaton is a network dynamics model featuring discrete time, space, and state, where spatial interactions and temporal causality remain localized [9,29]. Its structure generally comprises a cellular space, individual cells, a set of possible cell states, a neighborhood, and evolutionary rules governing state transitions, and can be represented by the quadruple $CA=(\Omega_{d},C,N,F)$. The symbol CA here designates the cellular automaton itself. The term $\Omega_{d}$ denotes the d-dimensional cellular space, which is essentially a discrete grid structure, usually one- or two-dimensional. A cell’s state is represented by C, a value that depends jointly on the cell’s own condition and on the conditions of its immediate neighbors. The neighborhood is denoted by N, consisting of those cells that can affect the central cell in the subsequent time step. Lastly, F accounts for the dynamical evolution rules, which prescribe how cells transition between states over time.

Edges in the complex network indicate business relationships and information exchange, while food firms are treated as its nodes. A scale-free network is thus formed and adopted as the cellular space. Each food firm constitutes a single cell, and other food firms associated with a given firm are its neighboring cells. Following Wang et al. and Hassan et al. [34,35], and in view of the actual state of risk resonance among food firms, the cells are grouped into four categories:

(1) Susceptible-state firms (S): These have not yet experienced risk resonance but, under the combined influence of media communication strategy, government regulatory policies, and the heterogeneity of food firms, are susceptible to being influenced by infected-state firms and may subsequently develop risk resonance.
(2) Latent-state firms (H): Firms that have been affected by risk resonance but do not yet exhibit resonant characteristics. After a certain period, they may transform into infected-state firms or directly into immune-state firms.
(3) Infected firms (I): Food firms that have already experienced risk resonance, are highly sensitive to food safety incidents, and are capable of transmitting risks to associated firms.
(4) Immune-state firms (R): Food firms possessing strong risk resilience, capable of remaining unaffected by infected-state firms or transitioning from an infected state through risk prevention and control measures. They may also revert to a susceptible state due to the failure of immunity.

The four state counts of food firms in the spatial–temporal association network are represented by s, h, i, and r. k is the maximum degree of food firms, and N is the network size. $S_{k}(t)$, $H_{k}(t)$, $I_{k}(t)$, and $R_{k}(t)$ represent the densities of susceptible, latent, infected, and immune firms that have degree k at time t, where $S_{k}(t)+H_{k}(t)+I_{k}(t)+R_{k}(t)=1$. ($0\leq S_{k}(t),H_{k}(t),I_{k}(t),R_{k}(t)\leq 1$). At time t, we assume the connection probability between a susceptible firm and an infected firm is $\Theta_{1}(t)$.

#### 3.1.2. Dynamical Evolution Rule

Drawing on the work of Pereira et al. and Michele et al. [36,37], we incorporate cellular automata to better capture how the number of infected neighbors numerically affects the infection rate, thereby improving the model’s alignment with the real-world phenomenon of corporate resonance diffusion of food safety risk. The cell state set C is (0, 1, 2, 3), with 0 representing a susceptible firm, 1 a latent-state firm, 2 an infected firm, and 3 an immune firm. Through the introduction of the classical SHIRS infectious disease dynamics equations, and by considering the mutual influence of neighboring cell states, we express the transition rules between the four firm states as follows:

(1) Under a food safety incident, a susceptible firm moves from state S to state H with probability $p=1-{\left(1-\tau \right)}^{\inf}$, provided that infected firms exist among its neighboring nodes. The variable inf here stands for the count of infected firms in the neighborhood.
(2) After a food safety incident occurs, most latent-state firms shift to the infected state with probability δ, provided that media influence is substantial and reporting preference is extreme, and given the combined effect of corporate influence and corporate risk preference. Meanwhile, a smaller portion of latent-state firms, driven by strong corporate social responsibility and extreme risk preference, move directly to the immune state with probability η, as they internalize food safety risk for self-regulation purposes.
(3) When government penalties are severe and regulatory information transparency is high, infected firms transition to immune firms with a probability of μ. When government penalties and regulatory information transparency are low, and media disclosure intensity is also low, immune firms revert to susceptible firms with a probability of γ.

**Figure 2:**
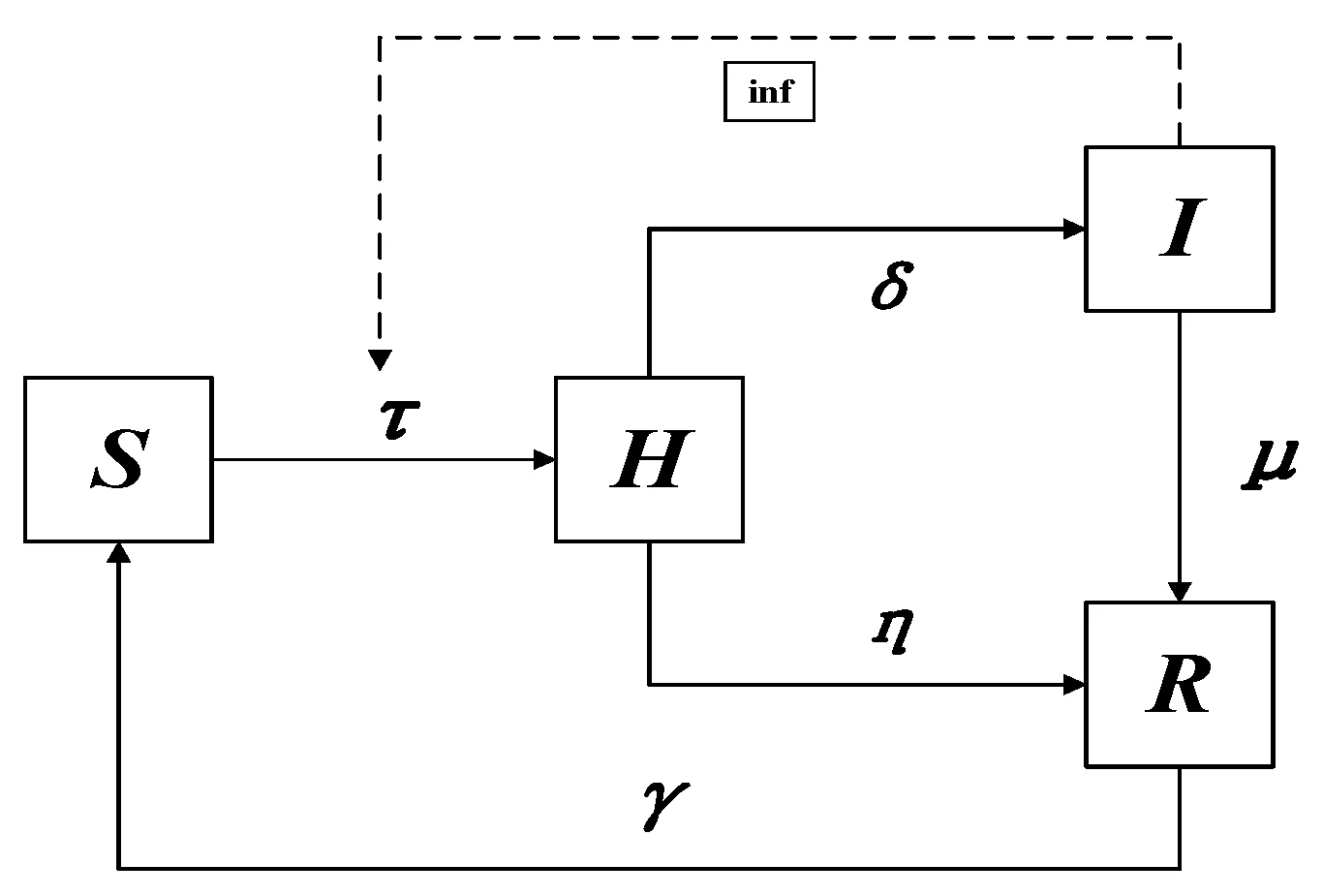
The CA-SHIRS model of corporate resonance diffusion of food safety risk.

Figure 2 presents the CA-SHIRS model of corporate resonance diffusion of food safety risk, which is constructed based on the infectious disease dynamical models established above.

The infection probability of corporate resonance of food safety risk among firms is defined as follows, based on the methodology of Wang et al. [13] and the integration of the Gilpin–Ayala information diffusion model with behavioral effect functions.

(1) $$\tau =\frac{15}{2}e^{\frac{-\omega }{\sigma }}\sqrt{1-\phi }(1-\varepsilon^{2})(1-e^{\frac{\theta }{\xi }})(\varphi -\varphi^{2}){\left(\rho -0.5\right)}^{2}$$

In this context, $f(\sigma ,\varphi ,\omega )=5e^{\frac{-\omega }{\sigma }}(\varphi -\varphi^{2})$ represents the food firm heterogeneity function. $\sigma \left(0\leq \sigma \leq 1\right)$ denotes corporate influence [38,39]. A higher value of σ indicates that a food firm has a greater ability to influence changes in the status of other food firms through its market position, network relationships and reputation. $\varphi \left(0\leq \varphi \leq 1\right)$ represents corporate risk preference [40]. φ=0.5 indicates that food firms tend to adopt a mainstream, bandwagon strategy when facing food safety risk, which is the most common approach. φ approaching 1 indicates that food firms tend to adopt a risk-taking strategy when facing risks. φ approaching 0 indicates that food firms tend to adopt a conservative strategy when facing risks. $\omega \left(0\leq \omega \leq 1\right)$ represents corporate social responsibility [41]. ω indicates that the higher the value, the stronger the food firm’s sense of social responsibility and the greater its willingness to operate in compliance. $h(\theta ,\rho ,\xi )=3(1-e^{\frac{-\theta }{\xi }}){\left(\rho -0.5\right)}^{2}$ is the media communication strategy function. $\theta \left(0\leq \theta \leq 1\right)$ represents media influence [42]. θ indicates that the higher the value, the greater the media visibility, the wider the communication range, and the faster the resonance-driven diffusion. $\rho \left(0\leq \rho \leq 1\right)$ represents media reporting preference [43,44]. ρ=0.5 represents neutral and rational media coverage. ρ approaching 1 indicates whitewashing by the media, where overly positive coverage raises questions of credibility and thus stimulates the rapid flow of risk information. ρ approaching 0 indicates a tendency towards negative media coverage. $\xi \left(0\leq \xi \leq 1\right)$ represents media information disclosure intensity [45,46]. A higher ξ corresponds to greater authenticity and timeliness in media coverage, along with more detailed reporting. Furthermore, $g(\phi ,\varepsilon )=\frac{\sqrt{1-\phi }(1-\varepsilon^{2})}{2}$ represents the government regulatory strategy function. $\phi \left(0\leq \phi \leq 1\right)$ denotes government penalty intensity [47]. ϕ indicates that a higher value signifies stricter penalties imposed by the government on non-compliant food firms. $\varepsilon \left(0\leq \varepsilon \leq 1\right)$ represents government regulatory information transparency [48]. ε indicates that a higher value signifies greater transparency in regulatory information, thereby reducing information asymmetry among food firms. Table 1 presents the detailed interpretations and benchmark values of parameters adopted in this study.

Based on the mechanism of corporate resonance diffusion of food safety risk and the state transition rules between food firms, the CA-SHIRS model is formulated as follows:

(2) $$\begin{array}{l} \left\{\begin{array}{l} \frac{dS_{k}(t)}{dt}=-k\tau S_{k}(t)\Theta (t)+\gamma R_{k}(t)\\ \frac{dH_{k}(t)}{dt}=k\tau S_{k}(t)\Theta (t)-(\delta +\eta )H_{k}(t)\\ \frac{dI_{k}(t)}{dt}=\delta H_{k}(t)-\mu I_{k}(t)\\ \frac{dR_{k}(t)}{dt}=\mu I_{k}(t)+\eta H_{k}(t)-\gamma R_{k}(t) \end{array}\right. \end{array}$$

#### 3.1.3. Threshold Analysis of Corporate Resonance Diffusion of Food Safety Risk

The right-hand side of the system of differential Equation (2) is set to zero. Once convergence to a steady state is achieved by the spatial–temporal association network of food firms, the steady-state densities associated with susceptible, latent, infected, and immune firms of degree k are then derived as follows:

(3) $$\left\{\begin{array}{l} S_{k}^{*}(t)=\frac{\gamma \mu (\delta +\eta )}{\gamma \mu (\delta +\eta )+\gamma \mu \tau k\Theta (t)+\gamma \delta \tau k\Theta (t)+\mu \left(\delta +\eta \right)\tau k\Theta (t)}\\ H_{k}^{*}(t)=\frac{\gamma \mu \tau k\Theta (t)}{\gamma \mu (\delta +\eta )+\gamma \mu \tau k\Theta (t)+\gamma \delta \tau k\Theta (t)+\mu \left(\delta +\eta \right)\tau k\Theta (t)}\\ I_{k}^{*}(t)=\frac{\gamma \delta \tau k\Theta (t)}{\gamma \mu (\delta +\eta )+\gamma \mu \tau k\Theta (t)+\gamma \delta \tau k\Theta (t)+\mu \left(\delta +\eta \right)\tau k\Theta (t)}\\ R_{k}^{*}(t)=\frac{\mu \left(\delta +\eta \right)\tau k\Theta (t)}{\gamma \mu (\delta +\eta )+\gamma \mu \tau k\Theta (t)+\gamma \delta \tau k\Theta (t)+\mu \left(\delta +\eta \right)\tau k\Theta (t)} \end{array}\right.$$

Furthermore, since $\Theta (t)=\frac{1}{<k>}\sum_{k}kP(k)I_{k}(t)$, where <k> denotes the mean connectivity of the spatial–temporal association network of food firms, and given that $<k>=\sum_{k}kP(k)$ and $<k^{2}>=\sum_{k}k^{2}P(k)$, we further obtain:

(4) $$\Theta (t)=\frac{1}{<k>}\sum_{k}kP(k)\frac{\gamma \delta \tau k\Theta (t)}{\gamma \mu (\delta +\eta )+\gamma \mu \tau k\Theta (t)+\gamma \delta \tau k\Theta (t)+\mu \left(\delta +\eta \right)\tau k\Theta (t)}$$

Let Θ=Θ(t). The above equation has the trivial solution Θ=0. If Equation (4) has a non-trivial solution, i.e., Θ≠0, then the necessary condition is:

(5) $$\frac{d}{d\Theta }\left(\frac{1}{<k>}\sum_{k}kP(k)\frac{\gamma \delta k\tau \Theta (t)}{\gamma \mu (\delta +\eta )+\gamma \mu \tau k\Theta (t)+\gamma \delta \tau k\Theta (t)+\mu \left(\delta +\eta \right)\tau k\Theta (t)}\right)\left|\Theta =0\geq 1\right.$$

That is, $\frac{1}{<k>}\sum_{k}kP(k)\frac{\delta k\tau }{\mu \left(\delta +\eta \right)}\geq 1$. Thus, the basic reproduction number $R_{0}$, under the interaction of food firm heterogeneity, media communication strategy, and government regulatory strategy, for the corporate resonance diffusion of food safety risk is:

(6) $$R_{0}=\frac{\delta \tau \sum_{k}k^{2}P(k)}{\mu (\delta +\eta )\sum_{k}kP(k)}=\frac{15e^{\frac{-\omega }{\sigma }}\sqrt{1-\phi }(1-\varepsilon^{2})(1-e^{\frac{\theta }{\xi }})(\varphi -\varphi^{2}){\left(\rho -0.5\right)}^{2}\delta \sum_{k}k^{2}P(k)}{2\mu (\delta +\eta )\sum_{k}kP(k)}$$

In a scale-free network, $<k>=2m,<k^{2}>=\sum_{k}k^{2}P(k),P(k)=\frac{2m^{2}}{k^{3}}$, with the network’s maximum node degree set to $k_{\max}$. When the total count of network nodes goes to infinity, $k_{\max}=mN^{2}$, $<k^{2}>=2m^{2}\ln(\frac{k_{\max}}{m})$. Therefore, after simplification, the basic reproduction number $R_{0}$ for the corporate resonance diffusion of food safety risk is:

(7) $$\begin{array}{l} R_{0}=\frac{30e^{\frac{-\omega }{\sigma }}\sqrt{1-\phi }(1-\varepsilon^{2})(1-e^{\frac{\theta }{\xi }})(\varphi -\varphi^{2}){\left(\rho -0.5\right)}^{2}\delta \sum_{k}k^{2}P(k)}{\mu (\delta +\eta )\sum_{k}kP(k)}\\ =\frac{30e^{\frac{-\omega }{\sigma }}\sqrt{1-\phi }(1-\varepsilon^{2})(1-e^{\frac{\theta }{\xi }})(\varphi -\varphi^{2}){\left(\rho -0.5\right)}^{2}\delta m\ln(N)}{\mu (\delta +\eta )} \end{array}$$

#### 3.1.4. Theoretical Analysis of Corporate Resonance Diffusion of Food Safety Risk

**Definition** **1.** *For the early stage of corporate resonance diffusion of food safety risk,* $R_{0}$* is the average number of susceptible firms infected by a single infected firm before it recovers. This is the basic reproduction number within the spatial–temporal association network of food firms. Typically,* $R_{0}=1$ *serves as a critical threshold for determining whether corporate resonance diffusion ceases. When* $R_{0}<1$*, the probability of corporate resonance transmission is low and cannot trigger a chain reaction. The corporate resonance diffusion of food safety risk will not continue and will gradually cease. When* $R_{0}>1$*, susceptible firms will be infected and transform into latent and infected firms. Furthermore, a higher* $R_{0}$ *implies a greater transmission likelihood in the spatial–temporal association network of food firms.*

**Proposition** **1.** *The corporate resonance diffusion of food safety risk has a unique risk equilibrium point* $\Theta^{*}$, and $\Theta^{*}=0$.

From the perspective of epidemic dynamics, susceptible, latent, infected, and immune states can coexist at equilibrium in heterogeneous networks. The equilibrium is unique under the setting of this model. The system will eventually reach a stable state regardless of initial conditions [49,50,51].

**Proof.**  Let $W_{k}(\Theta )=\frac{k^{2}P(k)}{<k>}\sum_{k}\frac{\gamma \delta k\tau \Theta (t)}{\gamma \mu (\delta +\eta )+\gamma \mu \tau k\Theta (t)+\gamma \delta \tau k\Theta (t)+\mu \left(\delta +\eta \right)\tau k\Theta (t)}$. Since $W_{k}\prime (\Theta )=\frac{k^{2}P(k)}{<k>}\sum_{k}\frac{\delta \tau k}{\mu (\delta +\eta )}\times \frac{{\left[\gamma \mu (\delta +\eta )\right]}^{2}}{{\left\{\gamma \mu (\delta +\eta )+\tau k\Theta (t)[\gamma \mu +\gamma \delta +\mu (\delta +\eta )]\right\}}^{2}}>0$ and $W_{k}\prime \prime (\Theta )<0$, it follows that $W_{k}(\Theta )$ is a monotonically increasing concave function of Θ. Furthermore, since $W_{k}(1)=\frac{k^{2}P(k)}{<k>}\sum_{k}\frac{\gamma \delta k\tau }{\gamma \mu (\delta +\eta )+\gamma \mu \tau k+\gamma \delta \tau k+\mu \left(\delta +\eta \right)\tau k}<\frac{kP(k)}{<k>}\sum_{k}\frac{\gamma \delta k\tau }{\gamma \mu (\delta +\eta )+\gamma \mu \tau k+\gamma \delta \tau k+\mu \left(\delta +\eta \right)\tau k}=1$ and $W_{k}(0)=0$, it follows that $W_{k}(\Theta )=\frac{k^{2}P(k)}{<k>}\sum_{k}\frac{\gamma \delta k\tau \Theta (t)}{\gamma \mu (\delta +\eta )+\gamma \mu \tau k\Theta (t)+\gamma \delta \tau k\Theta (t)+\mu \left(\delta +\eta \right)\tau k\Theta (t)}$ has a unique fixed point $\Theta^{*}$ on [0,1], and $\Theta^{*}>0$. Thus, Proposition 1 is proven. □

Hence, corporate resonance is always present with a nonzero probability in the spatial–temporal association network of food firms when it converges to a risk equilibrium. The network then contains firms in all four states: susceptible, latent, infected, and immune. In such a setting, food firms must remain vigilant, prevent influence from neighboring firms, and prepare for corporate resonance responses.

**Proposition** **2.** *The threshold* $R_{0}$* for the corporate resonance diffusion of food safety risk is negatively associated with corporate social responsibility* ω*. A positive relationship holds between corporate influence* σ *and the threshold* $R_{0}$ *for the corporate resonance diffusion of food safety risk.*

Food firms with higher social responsibility tend to internalize risk externalities and become less active in spreading risks [52]. Food firms with high influence hold key positions in the network and can spread risk signals to a wider range of connected firms [53].

**Proposition** **3.** *Government penalty intensity* ϕ*, government regulatory information transparency* ε*, and media information disclosure intensity* ξ *are inversely correlated with the threshold* $R_{0}$ *for the corporate resonance diffusion of food safety risk. The threshold* $R_{0}$ *for corporate resonance diffusion in food safety risk rises with media influence* θ*, indicating a positive relation.*

Tougher punishment and higher transparency can raise the cost of non-compliance and reduce information asymmetry [54,55]. These changes can slow the spread of risks. Media outlets with wide reach can speed up the transmission of risk signals to the public [56].

**Proof.**  The first-order derivative of Equation (7) with respect to ω is: $\frac{\partial R_{0}}{\partial \omega }=-\frac{\frac{30}{\sigma }e^{\frac{-\omega }{\sigma }}\sqrt{1-\phi }(1-\varepsilon^{2})(1-e^{\frac{\theta }{\xi }})(\varphi -\varphi^{2}){\left(\rho -0.5\right)}^{2}\delta m\ln(N)}{\mu (\delta +\eta )}<0$. Thus, an inverse relationship exists between corporate social responsibility ω and the corporate resonance diffusion threshold $R_{0}$ for food safety risk. $\frac{\partial R_{0}}{\partial \sigma }=\frac{\frac{30\omega }{\sigma^{2}}e^{\frac{-\omega }{\sigma }}\sqrt{1-\phi }(1-\varepsilon^{2})(1-e^{\frac{\theta }{\xi }})(\varphi -\varphi^{2}){\left(\rho -0.5\right)}^{2}\delta m\ln(N)}{\mu (\delta +\eta )}>0$. The corporate resonance diffusion threshold for food safety risk $R_{0}$ monotonically increases with corporate influence σ.

By similar reasoning, we obtain $\frac{\partial R_{0}}{\partial \phi }<0,\frac{\partial R_{0}}{\partial \varepsilon }<0,\frac{\partial R_{0}}{\partial \xi }<0,\frac{\partial R_{0}}{\partial \theta }>0$. Propositions 2 and 3 are thus proven. □

**Proposition** **4.** *The threshold for the corporate resonance diffusion of food safety risk* $R_{0}$ *is a convex function of firm risk preference* φ *that is first monotonically increasing and then monotonically decreasing. The threshold for the corporate resonance diffusion of food safety risk* $R_{0}$ *is a concave function of media reporting preference* ρ *that is first monotonically decreasing and then monotonically increasing.*

Food firms with moderate risk preference are easily affected by peer behavior and serve as important nodes for risk transmission [57]. Extreme media reports can heighten information uncertainty. Neutral media reports can provide rational guidance and curb excessive market reactions [58,59].

**Proof.**  Likewise, differentiating Equation (7) twice with respect to the firm risk preference φ yields $\frac{\partial R_{0}}{\partial \varphi }=\frac{30e^{\frac{-\omega }{\sigma }}\sqrt{1-\phi }(1-\varepsilon^{2})(1-e^{\frac{\theta }{\xi }})(\varphi -2\varphi ){\left(\rho -0.5\right)}^{2}\delta m\ln(N)}{\mu (\delta +\eta )}$ and $\frac{\partial^{2}R_{0}}{\partial \varphi^{2}}=-\frac{60e^{\frac{-\omega }{\sigma }}\sqrt{1-\phi }(1-\varepsilon^{2})(1-e^{\frac{\theta }{\xi }}){\left(\rho -0.5\right)}^{2}\delta m\ln(N)}{\mu (\delta +\eta )}<0$. Therefore, we have:

(8) $$\begin{array}{l} \left\{\begin{array}{l} \frac{\partial R_{0}}{\partial \varphi }>0,\varphi <\frac{1}{2}\\ \frac{\partial R_{0}}{\partial \varphi }<0,\varphi >\frac{1}{2} \end{array}\right. \end{array}$$

By a similar line of reasoning, we obtain $\frac{\partial^{2}R_{0}}{\partial \rho^{2}}>0$. Proposition 4 is thus proven. □

Propositions 2–4 highlight the complex disturbances inherent in food firm interactions. To mitigate the likelihood and extent of corporate resonance diffusion of food safety risk, several measures are necessary. These include strengthening corporate social responsibility, curtailing media influence, raising government penalty intensity and regulatory transparency as appropriate, and keeping media reporting preference within reasonable bounds. Furthermore, given the dual nature of corporate risk preference, food firms should maintain their risk preference within reasonable limits when facing food safety risk resonance.

## 4. Results

### 4.1. Analysis of the Equilibrium Point for Corporate Resonance Diffusion of Food Safety Risk

**Figure 3:**
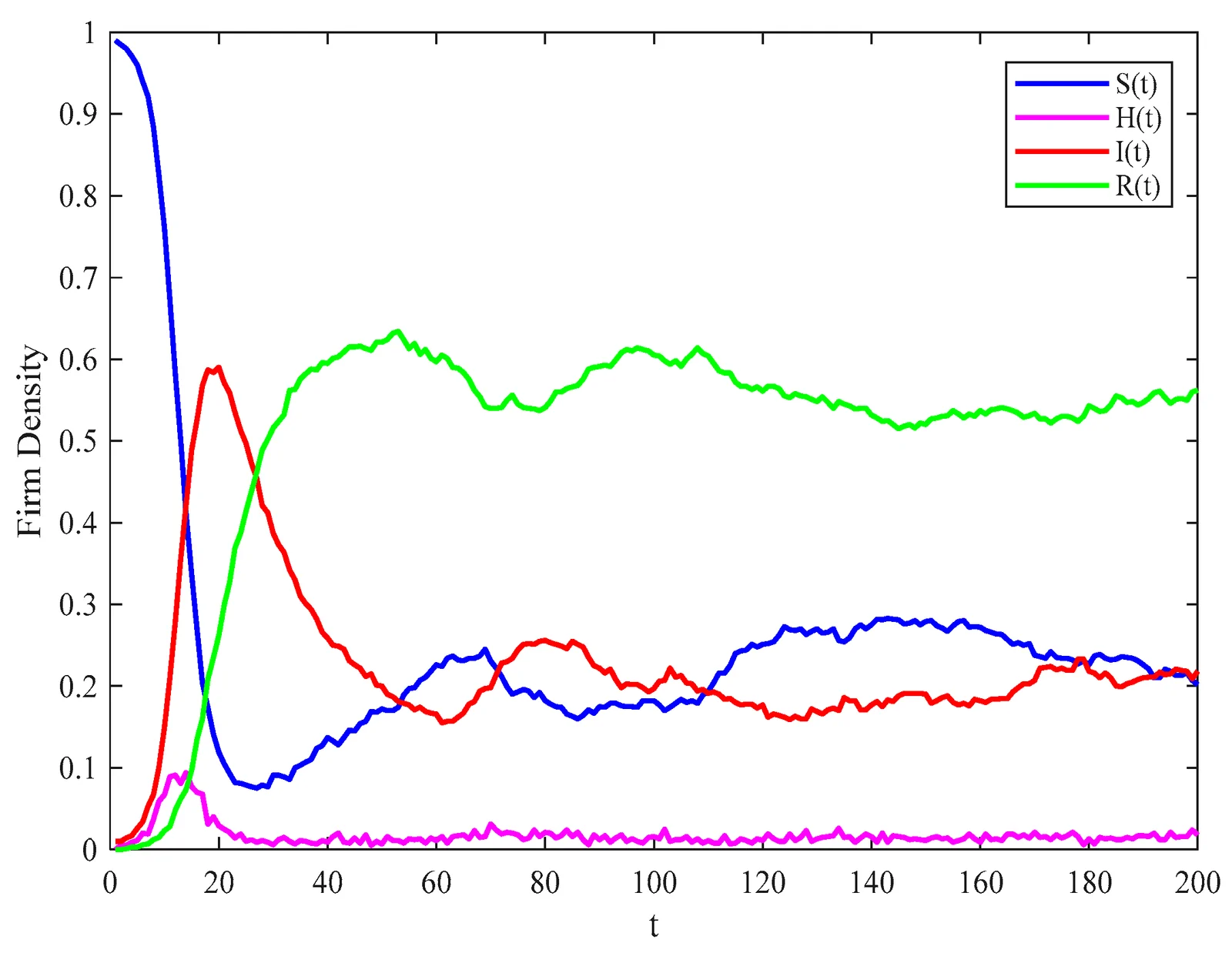
Evolutionary trends in the density of food firms in different states within the spatial–temporal association network of food firms.

Given that real-world spatial–temporal association network data for food firms often involves a vast array of supply chain collaborations, capital ties, regional competition and cooperation, and information exchange relationships, such data is not readily available. To facilitate research and ensure the generalizability of the findings, this section analyzes simulated networks. Assuming there are a total of N=1000 food firms in the spatial–temporal association network, with an initial number of connections per node of $m_{0}=3$ and a number of edges added upon the introduction of each new node of m=3. We generate a BA scale-free network to serve as the topology for the food firm network. Initially, one firm is assigned as infected (I). The other N−1 firms are set as susceptible (S). Furthermore, at the initial time, there are no food firms in the latent state (H) or the immune state (R), i.e., s(0)=N−1, h(0)=0, i(0)=1, and r(0)=0. We performed computational simulations using MATLAB R2025b (The MathWorks, Inc., Natick, MA, USA). For the case where parameters were fixed at τ=1,δ=0.5,η=0.02,μ=0.05,γ=0.05, Figure 3 provides our evaluation of the global stability of the risk equilibrium point, along with the evolutionary characteristics of firm scale across different states over diffusion time t.

Figure 3 displays the temporal variation in food firm densities across states within the spatial–temporal association network. Figure 3 illustrates that once a food safety incident occurs, the susceptible firm density falls sharply at first, then climbs gradually and eventually levels off. In contrast, both infected and latent firm densities surge to a peak and then subside progressively. The growth rate of the density of immune-state firms lags behind, eventually stabilizing. When a food firm is exposed to food safety risk during corporate resonance diffusion, the spatial–temporal association network’s small-world effect and clustering properties become relevant. Relatively minor food safety risk is capable of spreading quickly via networks like supply chains, capital linkages, and regional competition and cooperation. Thus, food safety incidents frequently generate effects that propagate quickly over a short time span and reach associated food firms. The affected food firms then pass the risk on to their associated counterparts, giving rise to a widespread, multi-regional, and cross-industry corporate resonance of food safety risk. During this process, latent-state and infected-state firms both show a pattern of first rising, then falling, and eventually leveling off. The reason is that after a certain period, latent-state firms have a certain probability of becoming infected-state firms. As the diffusion continues beyond a certain point, the speed of corporate resonance of food safety risk begins to decelerate. At this point, firm sizes in all four states settle into a relatively stable range, and the overall density across the spatial–temporal association network of food firms levels off without notable fluctuations.

**Figure 4:**
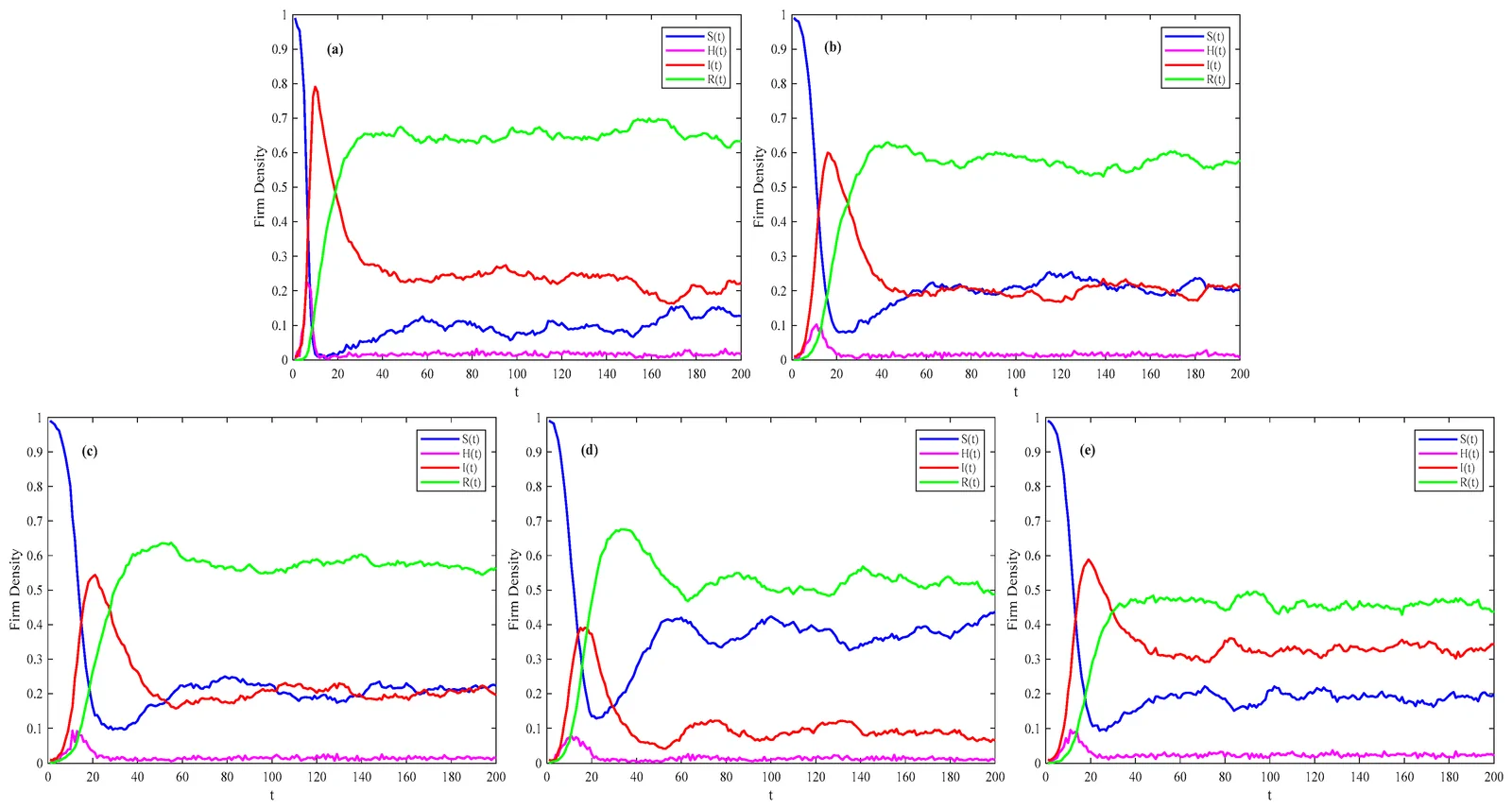
Evolutionary trends in the scale of food firms in different states during corporate resonance diffusion of food safety risk under different mechanism probabilities. (**a**) Increase infection probability; (**b**) Increase conversion probability; (**c**) Increase direct immune probability (**d**) Increase immune probability; (**e**) Increase immune failure probability.

We explored how corporate resonance diffusion of food safety risk evolves under various mechanism probabilities. These include direct immune probability η, infection probability τ, immune failure probability γ, immune probability μ, and conversion probability δ. Figure 4 displays the results, and Table 2 summarizes the numerical simulation parameter settings.

Various mechanism probabilities are considered in Figure 4, which presents the evolutionary trends of food firm scale across states during corporate resonance diffusion of food safety risk. After this process has been ongoing for a period, the spatial–temporal association network of food firms reaches a steady state, where the density in each state exhibits only slight fluctuations and remains broadly stable. Figure 4a shows that, with other parameters fixed, a higher infection probability allows the network to converge to a steady state more quickly. At the overall steady state, the number of susceptible firms decreases while latent firms increase. Additionally, the peak densities of latent and infected firms rise when the resonance proceeds. Thus, increasing the infection probability has two effects: it accelerates the network’s stabilization and magnifies the cross-regional impact of this resonance. These results are consistent with the observations of Michele et al. and Liang et al. [37,60] on how infection probability shapes risk transmission. Figure 4b indicates that, when other parameters are held constant, a higher conversion probability from latent to infected accelerates the network’s convergence to a steady state. In this case, the density of latent firms declines, while both the peak infected density and the steady-state scale increase. Therefore, raising the conversion probability both quickens the network’s convergence and intensifies the cross-regional and cross-industry spread of this resonance.

Figure 4e reveals that, with all other parameters held constant, an increase in immune failure probability leads to a reduction in immune-state firm density, whereas latent-state and infected-state firm densities rise once the network converges to a steady state. Thus, raising immune failure probability similarly accelerates the cross-regional and cross-sectoral spread of this resonance. Figure 4c further shows that, under fixed parameters, increasing direct immune probability in the latent state reduces the amplitude of firm-size fluctuations across all states and lowers the peak density of infected firms. Hence, raising the direct immune probability effectively curbs both the intensity and the extent of corporate resonance spread concerning food safety risk. Figure 4d illustrates that, with other parameters fixed, a rise in immune probability amplifies the fluctuations in firm scale across states. At the same time, the spatial–temporal association network of food firms requires more time to converge to a steady state. Once the network reaches steady state at this stage, susceptible firm density rises, whereas latent, infected, and immune firm densities all decline. Moreover, in the course of corporate resonance diffusion of food safety risk, peak values drop for both latent and infected firms. Thus, raising the immune probability produces two effects. It delays the network’s convergence to a steady state, and it also suppresses the propensity of this resonance to spread to other areas.

### 4.2. Spatial–Temporal Evolution Characteristics of Corporate Resonance Diffusion of Food Safety Risk

This section seeks to examine, via simulation, how various factors affect the corporate resonance diffusion of food safety risk under varying parameter settings. Accordingly, the analysis emphasizes the comparative patterns of parameter and outcome variations, rather than their absolute numerical values. To guarantee result reliability and stability, the network simulation was performed 20 times under constant parameters, and the mean density was derived from the simulation outcomes. Referring to the study by Michele et al. [37], the baseline parameter configuration is as follows: σ=0.8,φ=0.5,ω=0.2,θ=0.8,ρ=0.2,ξ=0.2,ϕ=0.2,ε=0.2.

The baseline parameter values in our simulations are not set uniformly to neutral levels but are deliberately configured to reflect a realistic high-risk scenario. Corporate influence and media influence are set at relatively high levels, capturing the dominant roles of large food firms and mainstream media in risk propagation. In contrast, corporate social responsibility, media information disclosure intensity, government penalty intensity, and government regulatory information transparency are set at low levels, reflecting the weak state of institutional risk-suppression mechanisms. Corporate risk preference is set at a moderate level to avoid biasing the diffusion process toward either extreme, while media reporting preference is set at a low level to capture the tendency toward negative coverage. This configuration establishes a baseline scenario characterized by weak suppression mechanisms and strong core diffusion agents, which provides a clear reference point for observing the independent effects of individual parameters through one-factor-at-a-time variations. The BA scale-free network parameters are selected to balance computational feasibility with statistical representativeness.

#### 4.2.1. Spatial–Temporal Evolutionary Characteristics of Corporate Resonance Diffusion of Food Safety Risk Under the Influence of Food Firm Heterogeneity

We conducted simulations using varying parameters for corporate influence, corporate risk preference, and corporate social responsibility to characterize the spatial–temporal evolution of corporate resonance diffusion of food safety risk under food firm heterogeneity.

**Figure 5:**
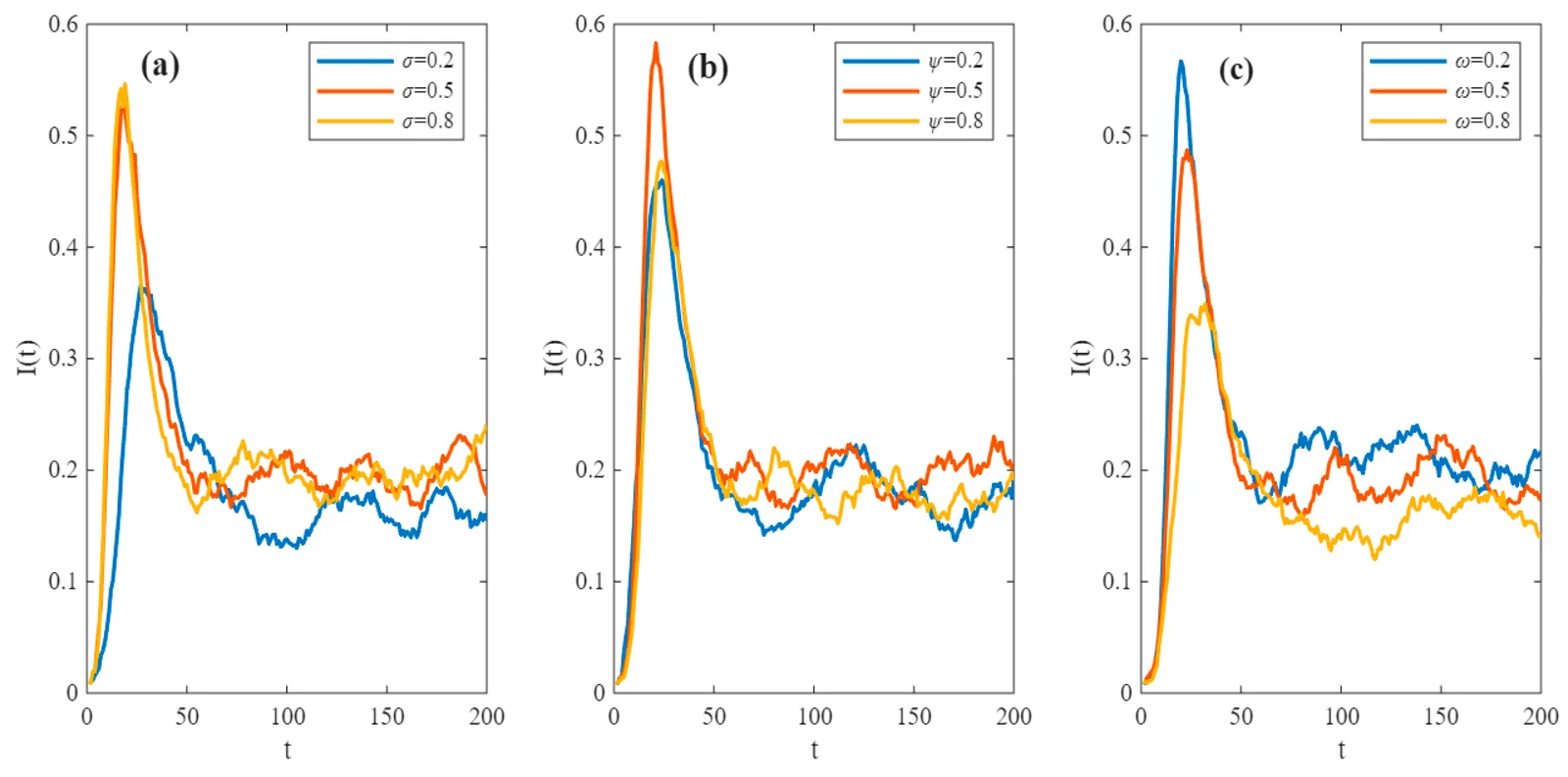
Spatial–temporal evolution characteristics of corporate resonance diffusion of food safety risk under the influence of food firm heterogeneity. (**a**) different values of corporate influence; (**b**) different values of corporate risk preference; (**c**) different values of corporate social responsibility.

Figure 5 depicts the time-dependent variation in infected firm density over time t as food safety risk spreads through corporate resonance. Different values are assigned to corporate influence, risk preference, and social responsibility, while other initial conditions stay unchanged. The figure further indicates that, under food firm heterogeneity, the infected firm count converges to a fixed value after a certain propagation duration t, and the network reaches a steady state. Figure 5a indicates that higher corporate influence leads to an increase in infected firm density. This in turn reduces the time needed for the network to attain a steady state, and also speeds up the cross-regional and cross-sectoral spread of this resonance. The underlying reason is that an increase in corporate influence enables food firms to occupy a more central position within supply chains and markets, making their risk signals easier for associated food firms to perceive and amplify, thereby facilitating the rapid cross-regional and cross-sectoral spread of this resonance.

Figure 5b shows that the impact of corporate risk preference on the density of infected firms exhibits an inverted U-shaped pattern. When corporate risk preference is at a moderate level, the density of infected firms reaches its maximum and the rate of diffusion is fastest. When corporate risk preference is extremely low or extremely high, that is, when firms are overly conservative or overly aggressive, the density of infected firms is relatively low. This is because food firms with moderate risk preference possess both sufficient risk sensitivity and a strong herd mentality. When faced with uncertainty, they are most likely to mimic the behavior of their peers, thereby amplifying the spread of risk. Food firms with extremely low risk preference react slowly, while those with extremely high risk preference are either desensitized to risk signals or act in a counterproductive manner. Neither group constitutes effective transmission nodes. Figure 5c demonstrates that higher corporate social responsibility lowers the density of infected firms in the spatial–temporal association network of food firms, while also decelerating the diffusion pace. This arises because corporate social responsibility enhances the propensity of food firms to adopt responsible measures when confronting food safety incidents. Such proactive responses curb the spillover of risk signals and thus lower the chance of food safety risk resonance propagating to other regions and industries.

These findings offer practical implications. High-influence food firms should be prioritized in regulatory monitoring. Firms with moderate risk preference are key transmission nodes and deserve special attention. Strengthening corporate social responsibility can help mitigate risk diffusion.

#### 4.2.2. Spatial–Temporal Evolution Characteristics of Corporate Resonance Diffusion of Food Safety Risk Under the Influence of Media Communication Strategy

To explore how media communication strategy shapes the spatial–temporal evolution of this resonance, we performed simulations with varying parameter values for media influence, media reporting preference, and media information disclosure intensity.

**Figure 6:**
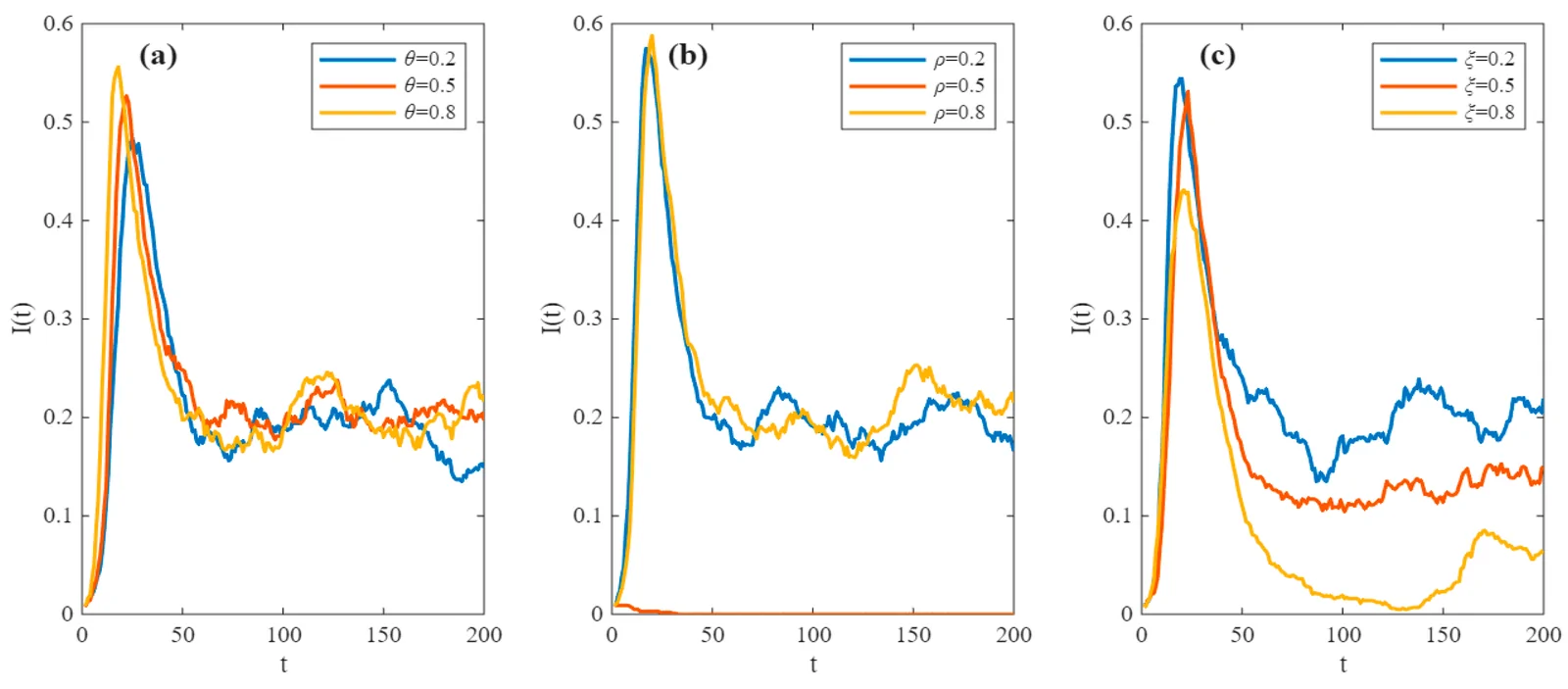
Spatial–temporal evolution characteristics of corporate resonance diffusion of food safety risk under the influence of media communication strategy. (**a**) different values of media influence; (**b**) different values of media reporting preference; (**c**) different values of media information disclosure intensity.

Figure 6 depicts how infected firm density varies over the diffusion time t of this resonance process, with all other initial parameters held fixed and media influence, media reporting preference, and media information disclosure intensity taking different values. Once this resonance persists for a sufficient period under media communication strategy, the network attains a steady state. Figure 6a reveals a positive association between media influence and the density of infected firms in the network. This is because high-influence media can disseminate risk signals more widely to a greater number of food firms, thereby accelerating the spread of risk within the network. Figure 6b shows that the impact of media reporting preference on the density of infected firms exhibits a positive U-shaped pattern. When media coverage tends to be extremely negative or extremely positive, the density of infected firms rises significantly. When media coverage tends to be neutral, the density of infected firms is at its lowest. This is because infection probability does not reach the threshold for corporate resonance diffusion of food safety risk when media coverage is neutral. Consequently, extreme reporting triggers strong reactions from food firms: negative coverage induces panic and herd behavior, while positive coverage, particularly whitewashing reports that do not align with facts, sparks skepticism and distrust. Both phenomena exacerbate the spread of risk signals. Neutral and rational reporting, however, helps reduce information uncertainty, providing food firms with a basis for rational judgment and thereby mitigating corporate resonance diffusion of food safety risk among firms.

Figure 6c shows a negative relationship between media information disclosure intensity and infected firm density within the spatial–temporal association network of food firms. This is because greater information disclosure intensity promotes transparency, lessens information asymmetry, and allows food firms to achieve a fuller grasp of the incident’s details. Consequently, it prevents overreactions and irrational decision-making caused by information gaps, thereby curbing the corporate resonance of food safety risk.

These results provide guidance for media governance. High-influence media platforms should be closely monitored during food safety incidents. Neutral reporting should be encouraged, while both negative and overly positive reporting should be discouraged. Improving media information disclosure can reduce information asymmetry and curb excessive market reactions.

#### 4.2.3. Spatial–Temporal Evolution Characteristics of Corporate Resonance Diffusion of Food Safety Risk Under the Influence of Government Regulatory Strategy

We conducted simulations using varying parameters for government penalty intensity and government regulatory information transparency to capture the spatial–temporal evolution of corporate resonance diffusion of food safety risk under government regulatory strategy.

**Figure 7:**
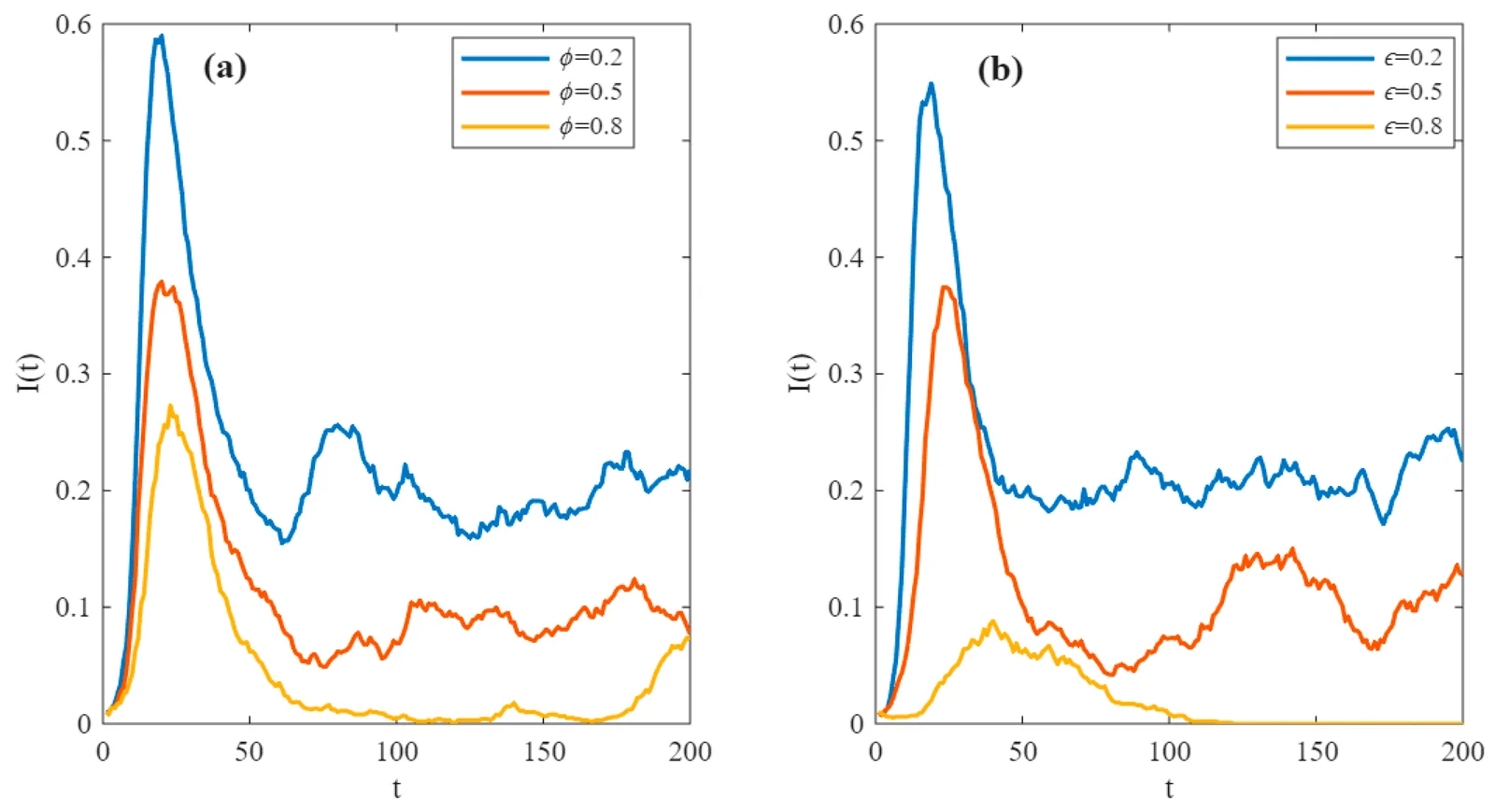
Spatial–temporal evolution characteristics of corporate resonance diffusion of food safety risk under the influence of government regulatory strategy. (**a**) different values of government penalty intensity; (**b**) different values of government regulatory information transparency.

Figure 7 depicts the temporal evolution of infected firm density over time t under varying levels of government penalty intensity and government regulatory information transparency. Other initial conditions are held constant. With government regulatory strategy in place, the spatial–temporal association network reaches a steady state after a certain duration of this resonance. Figure 7a,b further reveal that higher government penalty intensity and greater regulatory transparency correspond to lower infected firm density within the network. This is because stricter penalties generate a deterrent effect, curbing speculative behavior and the motivation for risk diffusion among food firms. Greater information transparency, in turn, reduces market information asymmetry, allowing food firms to detect risks earlier and implement countermeasures, which in turn suppresses the further diffusion of food safety risk resonance to other regions. It is worth noting that when government penalty intensity and the transparency of regulatory information are low, the density of infected firms rises significantly and the rate of diffusion accelerates, indicating that regulatory failure is a crucial catalyst for the spread of food safety risk resonance.

These results highlight the importance of government regulation. Stricter penalties deter non-compliant behavior. Greater regulatory transparency reduces information asymmetry and prevents overreaction. When both are weak, diffusion intensifies markedly.

#### 4.2.4. Spatial–Temporal Evolution Characteristics of Corporate Resonance Diffusion of Food Safety Risk Under the Interaction of Food Firm Heterogeneity, Media Communication Strategy and Government Regulatory Strategy

This section further explores the spatial–temporal evolution characteristics of this diffusion process. It does so by examining the interplay of food firm heterogeneity, media communication strategy, and government regulatory strategy.

**Figure 8:**
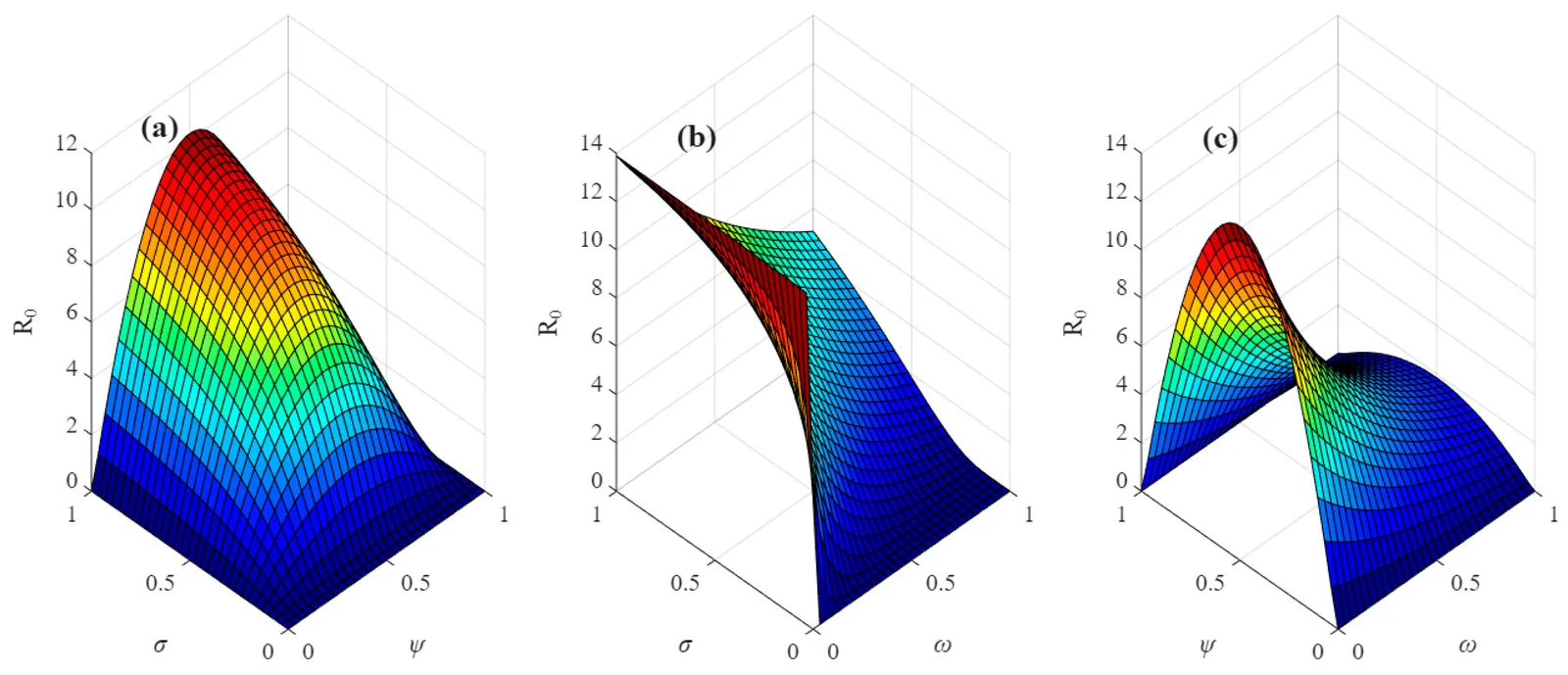
Spatial–temporal evolution characteristics of corporate resonance diffusion of food safety risk under the interaction of food firm heterogeneity. (**a**) The interaction between corporate influence and corporate risk preference; (**b**) The interaction between corporate influence and corporate social responsibility; (**c**) The interaction between corporate risk preference and corporate social responsibility.

Figure 8 demonstrates how interactions among multiple food firm heterogeneity traits influence the diffusion of this resonance, while other initial parameters remain fixed. Figure 8a reveals that, as corporate influence and corporate risk preference rise together, the intensity of corporate resonance diffusion of food safety risk initially surges and subsequently plunges. This mirrors the inverted U-shaped trend of corporate risk preference, while the increase in corporate influence significantly amplifies this trend. This indicates that the combination of high corporate influence and moderate risk preference is a key driver of corporate resonance diffusion. As shown in Figure 8b, when corporate influence and corporate social responsibility increase simultaneously, the intensity of corporate resonance diffusion of food safety risk exhibits a gradually decreasing trend. This indicates that corporate social responsibility exerts a stronger suppressive influence on corporate resonance diffusion than corporate influence exerts an amplifying one. Figure 8c illustrates that the intensity of this resonance exhibits a pattern of first rising and then falling when corporate risk preference and corporate social responsibility both increase in parallel. This mirrors the inverted U-shaped trend of corporate risk preference, while the increase in corporate social responsibility significantly attenuates this trend. The reason for this is that, when faced with the diffusion of this risk, food firms with strong social responsibility demonstrate a strong willingness to comply with regulations and are able to proactively suppress the spillover of risks. Therefore, maintaining corporate social responsibility at a relatively high level helps to suppress the intensity of corporate resonance diffusion of food safety risk.

**Figure 9:**
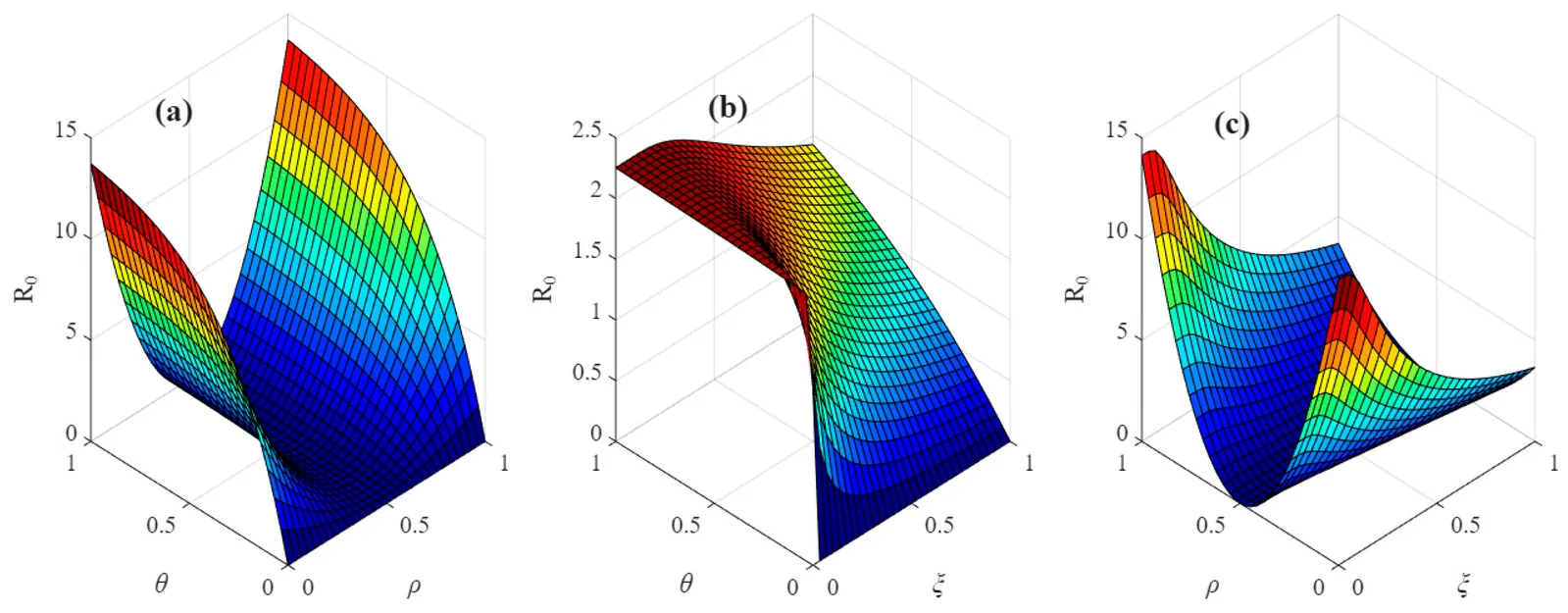
Spatial–temporal evolution characteristics of corporate resonance diffusion of food safety risk under the interaction of media communication strategy. (**a**) The interaction between media influence and media reporting preference; (**b**) The interaction between media influence and media information disclosure intensity; (**c**) The interaction between media reporting preference and media information disclosure intensity.

Figure 9 shows how media strategy factors interact to shape this resonance, while other initial parameters remain fixed. Figure 9a reveals that simultaneous increases in media influence and preference cause the intensity of corporate resonance to first rise, then dip, and then jump sharply. This is because, when media influence and media reporting preference are at relatively low levels, the risk-amplifying effect of media influence predominates. At moderate levels of both factors, the neutral and rational nature of media reporting preference exerts a moderating influence, causing the intensity of the diffusion of this risk to decline. For high levels, however, this situation reverses. The dual risk-amplifying effects of media influence and media reporting preference drive the intensity of corporate resonance diffusion of food safety risk upward sharply. Figure 9b reveals that as media influence and media information disclosure intensity increase simultaneously, the intensity of this resonance exhibits a decreasing trend. This indicates that the dampening effect of media information disclosure intensity on corporate resonance diffusion outweighs the reinforcing effect of media influence. As shown in Figure 9c, the intensity of corporate resonance diffusion of food safety risk first decreases sharply and then increases gradually as media reporting preference and media information disclosure intensity increase simultaneously. This mirrors the positive U-shaped trend observed in media reporting preference. However, as media information disclosure intensity gradually increases, its dampening effect on this trend also gradually strengthens. Therefore, maintaining media information disclosure intensity at a relatively high level helps to weaken the intensity of corporate resonance diffusion of this risk.

**Figure 10:**
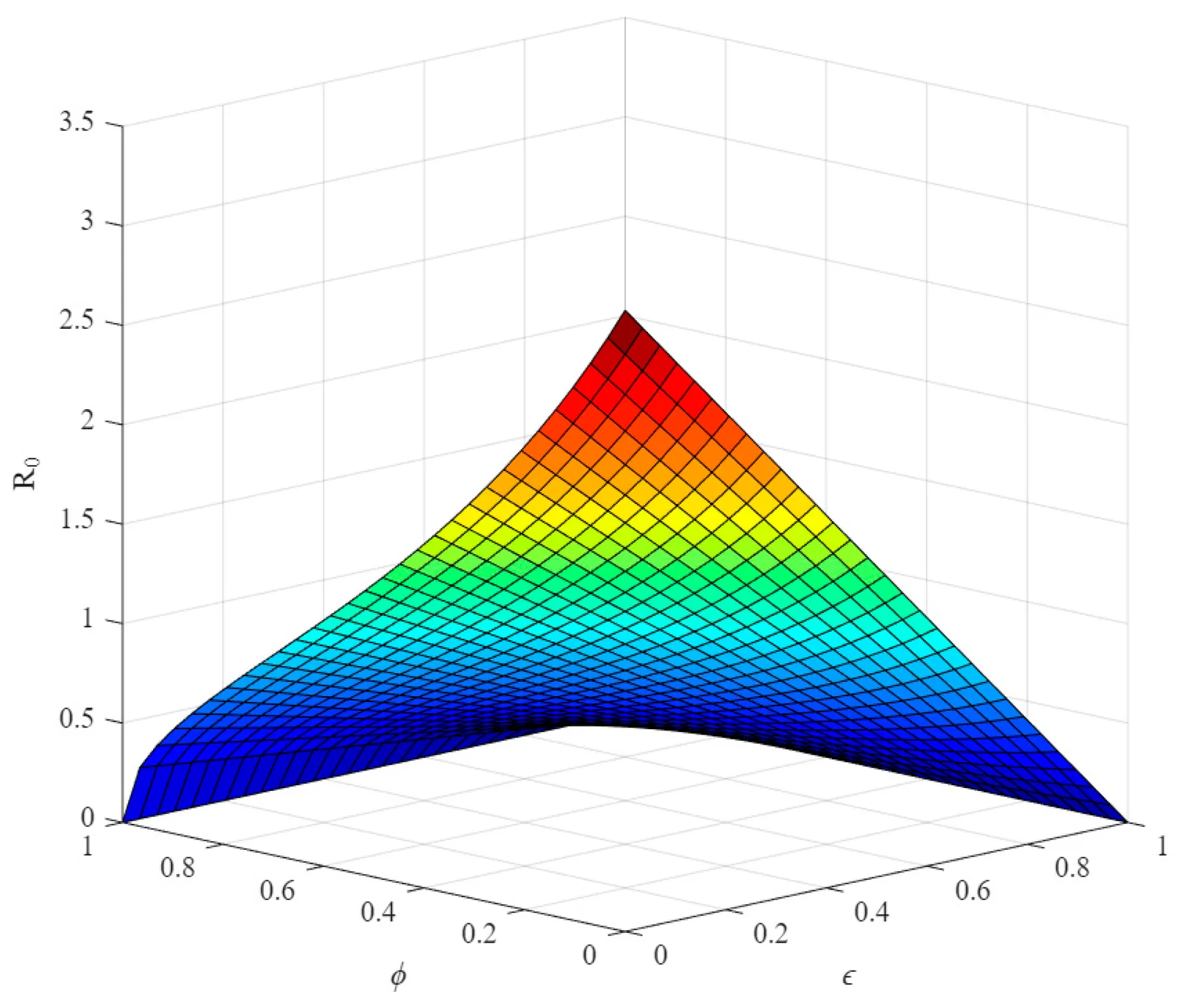
Spatial–temporal evolution characteristics of corporate resonance diffusion of food safety risk under the interaction of government regulatory strategy.

Figure 10 examines how the interaction of dual government regulatory strategy affects this resonance, given that all other starting parameters remain unchanged. The figure reveals that the intensity of this resonance drops sharply when government penalty intensity and regulatory transparency rise in tandem. This reflects that when severe government penalties and high regulatory information transparency act in concert, the effectiveness of resonant risk control far exceeds that of either factor alone. This finding further suggests that, to curb the corporate resonance diffusion of food safety risk, the government should impose stricter penalties and improve regulatory transparency in a timely manner. Doing so can effectively curb the speed and scale of cross-regional corporate resonance diffusion of food safety risk.

**Figure 11:**
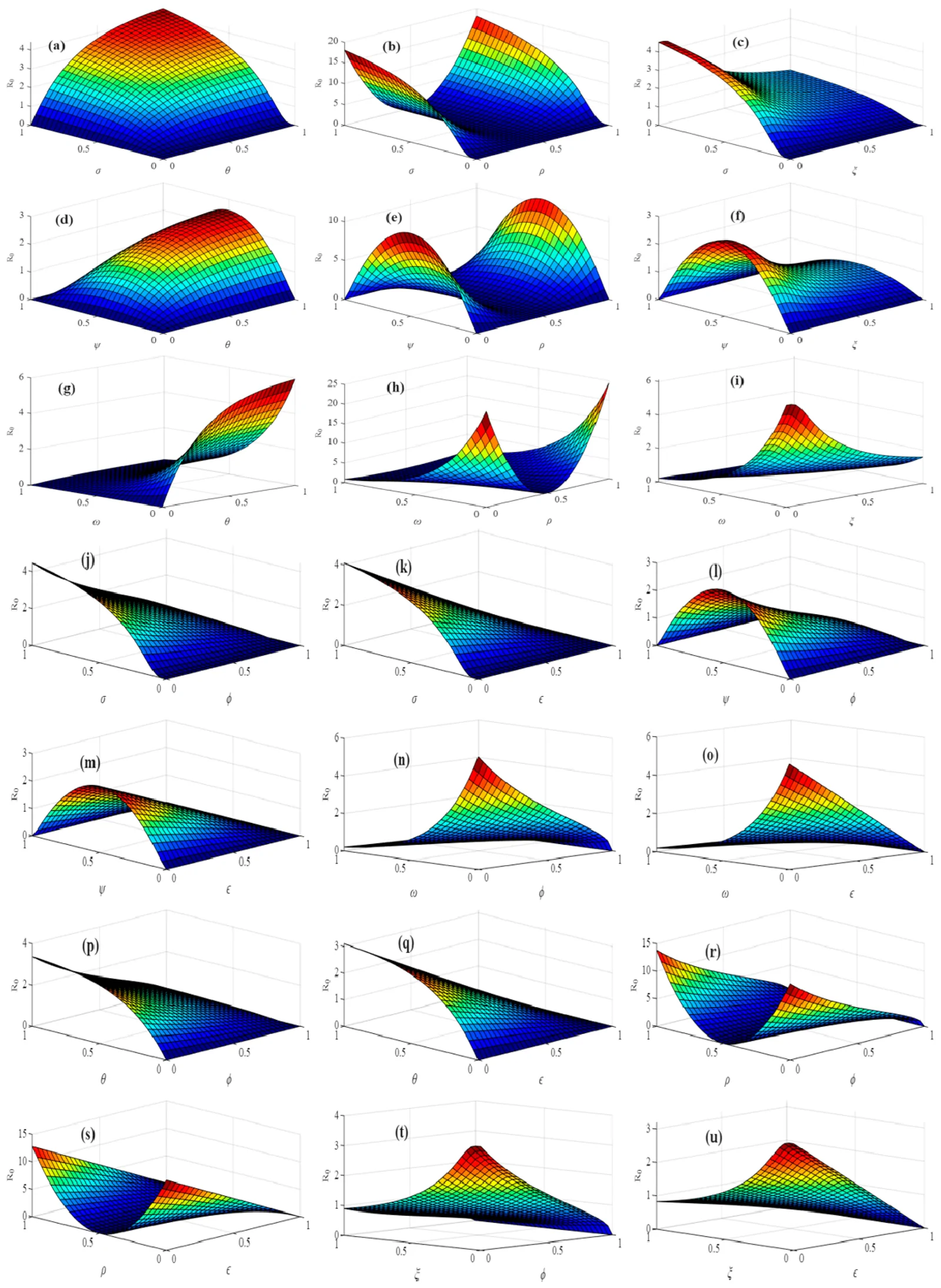
(**a**–**u**) Spatial–temporal evolution characteristics of corporate resonance diffusion of food safety risk under the interaction of food firm heterogeneity, media communication strategy and government regulatory strategy.

Figure 11 examines how food firm heterogeneity, media communication strategy, and government regulatory strategy interact to affect this resonance, with other initial parameters fixed. Figure 11a reveals that the intensity of corporate resonance diffusion surges sharply when corporate influence and media influence both increase together. This is because high-influence food firms occupy central positions within the network. Once their risk signals are amplified by high-influence media, they are highly likely to trigger panic across the entire market. Figure 11b shows that, with corporate influence and media reporting preference rising together, the intensity of this resonance diffusion follows a pattern of gradual ascent, followed by a gradual decline, and culminating in a sharp surge. This is because, when corporate influence and media reporting preference are at low levels, the resonant reinforcing effect of corporate influence dominates. The intensity of this resonance is moderated by the neutral and rational nature of media reporting preference when both factors are at moderate levels. In contrast, when both are elevated, the same intensity is propelled upward sharply by the dual risk-amplifying effects of media influence and media reporting preference.

As shown in Figure 11c,j,k, when corporate influence interacts with media information disclosure intensity, government penalty intensity and government regulatory information transparency respectively, the intensity of corporate resonance diffusion of food safety risk exhibits a pattern of initial increase followed by a decrease. This indicates that when the interactions between pairs of factors are at low to medium levels, the reinforcing effect of corporate influence on corporate resonance diffusion predominates. However, when these interactions reach medium to high levels, the dampening effects of media disclosure intensity, government penalty intensity, and government regulatory information transparency on corporate resonance diffusion outweigh the reinforcing effect of corporate influence. Furthermore, the dampening effects of government penalty intensity and government regulatory information transparency on corporate resonance diffusion are more pronounced.

A comparison of Figure 11d,f,l,m reveals that when corporate risk preference interacts with media influence, the intensity of this resonance diffusion regarding food safety risk first increases and then decreases. The same trend is observed when it interacts with media disclosure intensity, government penalty intensity, and government regulatory information transparency. This is because, when corporate risk preference is at a medium to low level, media influence and corporate risk preference exert a dual reinforcing effect on corporate resonance diffusion. In this case, the reinforcing effect of corporate risk preference outweighs the weakening effects of media disclosure intensity, government penalty intensity and government regulatory information transparency. When corporate risk preference is at a medium to high level, the intensity of media disclosure, government penalty intensity, and government regulatory information transparency, together with corporate risk preference, exert a dual-weakening effect on corporate resonance diffusion. However, the weakening effect of corporate risk preference on corporate resonance diffusion outweighs the reinforcing effect of media influence. Thus, keeping corporate risk preference at either a low or high level proves beneficial. It helps restrain this resonance diffusion within the spatial–temporal association network of food firms.

As depicted in Figure 11e, when corporate risk preference and media reporting preference increase in tandem, the intensity of corporate resonance diffusion of food safety risk exhibits a pattern of initial increase followed by a decrease, and subsequently increasing again before decreasing once more. This reflects the alternating influence of corporate risk preference and media reporting preference on corporate resonance diffusion of food safety risk. Among these, firms with moderate risk preference are most significantly affected by extreme media reporting.

A comparison of Figure 11h,I,n,o reveals that when corporate social responsibility interacts with media reporting preference, the intensity of media disclosure, government penalty intensity and government regulatory information transparency, respectively, the intensity of this resonance decreases sharply. This is because the simultaneous increase in corporate social responsibility, media information disclosure intensity, government penalty intensity, and government regulatory information transparency produces a synergistic inhibitory effect on corporate resonance diffusion, whereas the reinforcing effect of media reporting preference at medium to high levels is significantly smaller than the weakening effect of corporate social responsibility on corporate resonance diffusion. As shown in Figure 11g, when corporate social responsibility and corporate influence increase simultaneously, the intensity of this resonance diffusion first increases and then decreases. This is because, when corporate social responsibility is at a low to medium level, the reinforcing effect of corporate influence on corporate resonance diffusion is greater than the dampening effect of corporate social responsibility on corporate resonance diffusion, whereas when corporate social responsibility is at a medium to high level, the reinforcing effect of corporate influence on corporate resonance diffusion is far smaller than the dampening effect of corporate social responsibility on corporate resonance diffusion. Consequently, by maintaining a high level of corporate social responsibility and actively curbing the spillover of risks while strengthening compliance, companies can prevent this resonance.

Similarly, a comparison of Figure 11p,q,r,s reveals that when government penalty intensity and government regulatory information transparency increase in tandem with media influence, the intensity of this resonance diffusion first increases and then decreases. When government penalty intensity and government regulatory information transparency increase in tandem with media reporting preference, the intensity of corporate resonance diffusion of food safety risk first decreases, then increases, and then decreases again. It can thus be seen that when government penalty intensity and government regulatory information transparency are at relatively high levels, this resonance diffusion can be effectively suppressed. As shown in Figure 11t,u, when media information disclosure intensity increases in tandem with government penalty intensity and government regulatory information transparency, respectively, the intensity of this resonance diffusion decreases sharply. This indicates that the timely enhancement of penalty severity and regulatory transparency by the government, coupled with guidance for the media to increase the intensity of information disclosure, constitutes a key governance measure for controlling this resonance diffusion.

Comparing Figure 9 and Figure 11 further indicates that media reporting preference, when acting alone, exerts only a modest influence on the intensity of this resonance diffusion. In contrast, its interaction with food firm heterogeneity yields substantially more pronounced variations in corporate resonance diffusion. Stated differently, media reporting preference by itself carries limited weight in shaping corporate resonance diffusion of food safety risk. When combined with corporate influence and corporate risk preference, however, its negative repercussions on both the food firm sector and the broader food market are magnified, thereby widening the scope of corporate resonance across regions and industries. Extreme reporting can easily set off a chain of reactions among food firms and across regions in a short time, thus expanding the reach of corporate resonance. Therefore, in managing corporate resonance diffusion of food safety risk, efforts must go beyond merely moderating media reporting preference’s influence on firm perceptions. It is equally critical to raise government penalty intensity and improve regulatory information transparency to offset the adverse effects of extreme media coverage. Moreover, risk communication and regulatory measures should be tailored to the specific characteristics of food firm association networks in different localities, so as to effectively limit the speed and extent of cross-regional corporate resonance diffusion.

These interaction results offer actionable policy insights. The interplay between high-influence food firms and high-influence media can rapidly escalate risks and deserves close attention. Corporate social responsibility and media information disclosure work together to suppress diffusion. Government regulation plays an irreplaceable role and should be prioritized over relying solely on media self-discipline.

### 4.3. Robustness Test

The model is validated through a robustness analysis that systematically varies parameter configurations through three rounds of parameter adjustments across the three dimensions of food firm heterogeneity, media communication strategy, and government regulatory strategy. By examining all interactive combinations of the key factors, the robustness test confirms that the simulation results are not driven by specific parameter choices.

**Figure 12:**
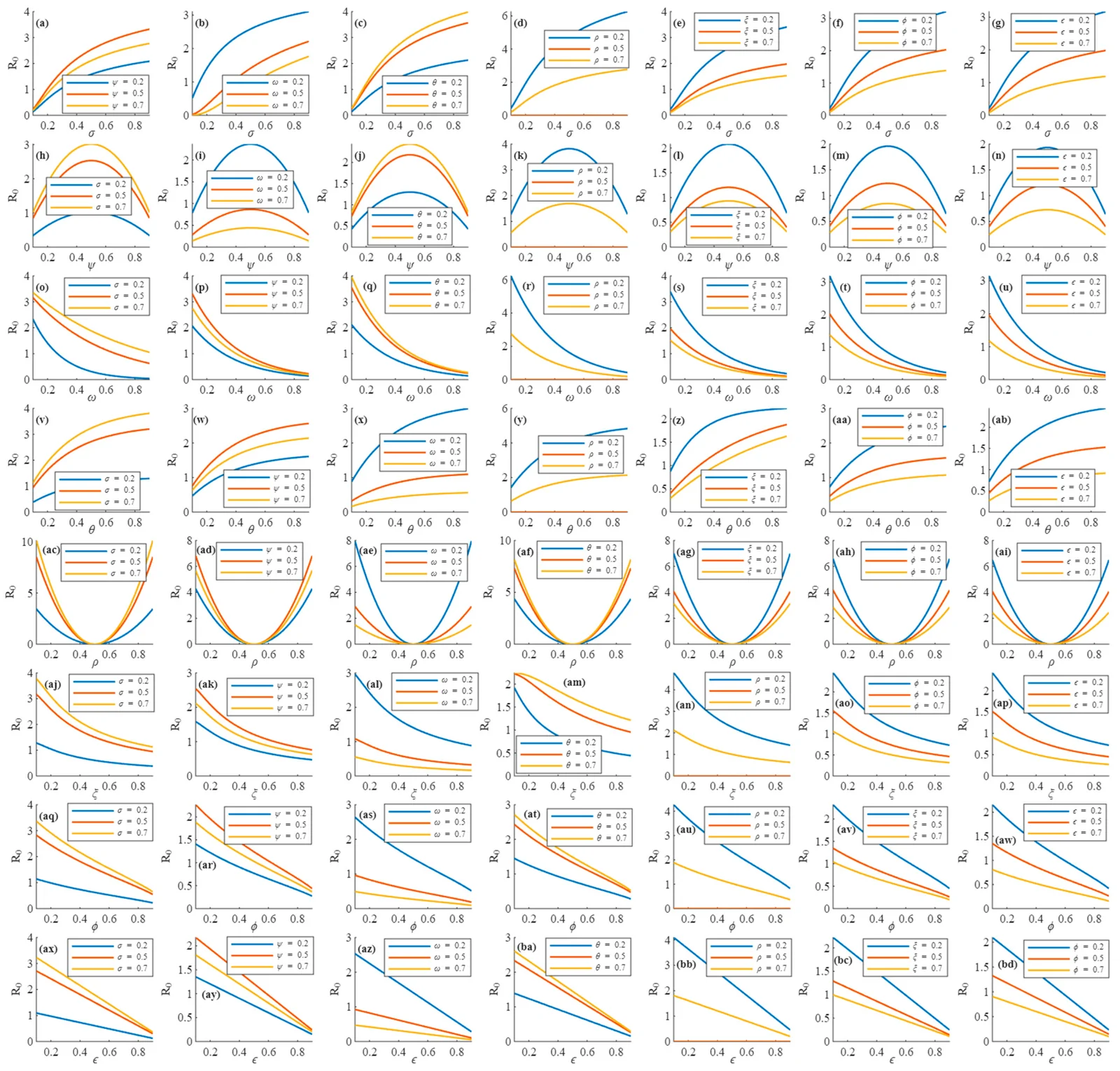
(**a**–**bd**) Robustness test of corporate resonance diffusion of food safety risk under the interaction of food firm heterogeneity, government regulatory strategy and media communication strategy.

The findings are presented in Figure 12. As shown in the figure, the influence of a given parameter on the probability trend of corporate resonance diffusion remains stable across different factor combinations, consistent with the analysis above. This confirms that the simulation-derived conclusions are highly robust. Figure 12 also corroborates the results obtained in Figure 8, Figure 9, Figure 10 and Figure 11, further reinforcing the robustness of both the constructed model and the simulation outcomes.

Direct empirical validation against real-world food safety events is not feasible due to limited data availability on food firm association networks. Consequently, the numerical results should be interpreted as directional indicators and comparative relationships rather than precise predictions. Nevertheless, the stability of the observed patterns across different parameter configurations supports the generalizability of our conclusions.

### 4.4. Result Discussion

This subsection discusses the key findings in relation to previous literature.

(1) Our finding that corporate influence amplifies resonance diffusion aligns with studies showing that high-degree nodes serve as key transmission hubs, and that enterprise influence increases the diffusion probability of unethical behavior among food firms. The inverted U-shaped relationship between risk preference and diffusion echoes the behavioral economics literature, where firms with moderate risk preference are most susceptible to peer influence and thus serve as the most effective transmission nodes. The inhibitory effect of corporate social responsibility is consistent with the finding that enterprise ethical climate can suppress the propagation of negative behaviors.
(2) Our results on media influence and reporting preference extend the findings of Wang et al. [13], who showed that media report tendency accelerates the spatial–temporal diffusion of food safety risk resonance. More importantly, we distinguish between media reporting preference and media information disclosure intensity which are two dimensions with opposite effects on diffusion.
(3) Our finding that stricter penalties and greater transparency suppress diffusion is consistent with Ma et al. [47], who advocated for stronger sanctions and improved information disclosure in food safety governance. Notably, when both penalties and transparency are weak, diffusion intensifies markedly, suggesting that regulatory failure is a key driver of resonance amplification.
(4) The interaction analyses reveal that corporate influence and media influence exert a dual reinforcing effect, while corporate social responsibility and media information disclosure intensity exert a synergistic inhibitory effect. These findings extend the existing literature, which has largely focused on isolated factors. The result that government regulation exerts a stronger inhibitory effect than the amplifying effect of media communication provides quantitative support for a government-led governance framework in food safety management.

## 5. Conclusions

In this paper, based on a comprehensive consideration of the interactions between food firm heterogeneity, media communication strategy and government regulatory strategy, a CA-SHIRS model for the corporate resonance diffusion of food safety risk was constructed from a space–time perspective, utilizing the SHIRS model and cellular automata. On this basis, the mechanism of this resonance diffusion was analyzed using the basic reproduction number, and numerical simulations were employed to reveal the dynamic evolutionary patterns of this corporate resonance diffusion across spatial–temporal dimensions. In summary, the principal findings derived from this study can be stated as presented below:

(1) Infection, conversion, and immune failure probabilities accelerate and expand the diffusion, while immune and direct immune probabilities help contain it. (2) The diffusion intensity is positively correlated with corporate influence and media influence, negatively correlated with corporate social responsibility, media information disclosure intensity, government penalty intensity, and government regulatory information transparency. It exhibits an inverted U-shaped relationship with corporate risk preference and a positive U-shaped relationship with media reporting preference. (3) Corporate influence and media influence exert a dual reinforcing effect, which government regulation effectively mitigates. Firms with moderate risk preference are most susceptible to extreme media coverage, and government regulation exerts a stronger inhibitory effect than the amplifying effect of media communication.

The findings offer practical implications for different stakeholders. (1) For regulatory authorities, priority should be given to monitoring high-influence food firms and strengthening both penalty intensity and information transparency. (2) For food business operators, strengthening social responsibility and maintaining risk preference at low or moderate levels can help contain risk spillover. (3) For media organizations, avoiding extreme reporting and improving information disclosure quality can effectively reduce excessive market reactions. (4) Importantly, the results highlight that government regulation plays an irreplaceable role and should be prioritized over reliance on media self-discipline in food safety governance.

These findings provide theoretical insights for different stakeholders to prevent and control corporate resonance diffusion of food safety risk while maintaining market stability. Nevertheless, the present work is confined to examining the mechanisms and spatial–temporal evolution of this diffusion process. It does not address the dynamic optimization of risk prevention and control strategies. Future research may extend this work in several directions. First, empirical calibration of the model using real-world food safety event data would enhance its predictive accuracy. Second, incorporating actual food business operator networks could replace the simulated network and improve external validity. Third, comparing the CA-SHIRS model with alternative approaches, such as agent-based models or machine learning frameworks, would help identify the most effective analytical tools. Beyond these extensions, future research will also explore the containment of this diffusion through corporate risk immunity mechanisms and government emergency intervention strategies.

## Figures and Tables

**Table 1:** Descriptions and numerical values of the model parameters.

Parameters	Descriptions	Baseline Values	Value Ranges
τ	Infection probability	1	[0, 1]
δ	Conversion probability	0.5	[0, 1]
η	Direct immune probability	0.02	[0, 1]
μ	Immune probability	0.05	[0, 1]
γ	Immune failure probability	0.05	[0, 1]
N	The total quantity of food firms in the network	1000	Positive Integer
m	The number of edges connected when each new node joins	3	Positive Integer
$m_{0}$	The number of connections of the initial node	3	Positive Integer
σ	Corporate influence	0.8	[0, 1]
φ	Corporate risk preference	0.5	[0, 1]
ω	Corporate social responsibility	0.2	[0, 1]
θ	Media influence	0.8	[0, 1]
ρ	Media reporting preference	0.2	[0, 1]
ξ	Media information disclosure intensity	0.2	[0, 1]
ϕ	Government penalty intensity	0.2	[0, 1]
ε	Government regulatory information transparency	0.2	[0, 1]

**Table 2:** Numerical variations in the CA-SHIRS model of corporate resonance diffusion of food safety risk under different mechanism probabilities.

τ	δ	η	μ	γ	Parameter Variations	Trend Chart
1	0.5	0.02	0.05	0.05	Unchanged	Figure 3
2	0.5	0.02	0.05	0.05	Increase infection probability	Figure 4a
1	0.7	0.02	0.05	0.05	Increase conversion probability	Figure 4b
1	0.5	0.05	0.05	0.05	Increase direct immune probability	Figure 4c
1	0.5	0.02	0.1	0.05	Increase immune probability	Figure 4d
1	0.5	0.02	0.05	0.1	Increase immune failure probability	Figure 4e

## Data Availability

The original contributions presented in this study are included in the article. Further inquiries can be directed to the corresponding author.
